# Quantifying and controlling bond multivalency for advanced nanoparticle targeting to cells

**DOI:** 10.1186/s40580-021-00288-1

**Published:** 2021-11-30

**Authors:** Elliot Y. Makhani, Ailin Zhang, Jered B. Haun

**Affiliations:** 1 Department of Materials Science and Engineering, University of California Irvine, Irvine, CA 92697 USA; 2 Department of Biomedical Engineering, University of California Irvine, 3107 Natural Sciences II, Irvine, CA 92697 USA; 3 Department of Chemical and Biomolecular Engineering, University of California Irvine, Irvine, CA 92697 USA; 4 Chao Family Comprehensive Cancer Center, University of California Irvine, Irvine, CA 92697 USA

**Keywords:** Nanoparticle, Targeting, Multivalent adhesions, Bond biophysics, Simulation

## Abstract

Nanoparticles have drawn intense interest as delivery agents for diagnosing and treating various cancers. Much of the early success was driven by passive targeting mechanisms such as the enhanced permeability and retention (EPR) effect, but this has failed to lead to the expected clinical successes. Active targeting involves binding interactions between the nanoparticle and cancer cells, which promotes tumor cell-specific accumulation and internalization. Furthermore, nanoparticles are large enough to facilitate multiple bond formation, which can improve adhesive properties substantially in comparison to the single bond case. While multivalent binding is universally believed to be an attribute of nanoparticles, it is a complex process that is still poorly understood and difficult to control. In this review, we will first discuss experimental studies that have elucidated roles for parameters such as nanoparticle size and shape, targeting ligand and target receptor densities, and monovalent binding kinetics on multivalent nanoparticle adhesion efficiency and cellular internalization. Although such experimental studies are very insightful, information is limited and confounded by numerous differences across experimental systems. Thus, we focus the second part of the review on theoretical aspects of binding, including kinetics, biomechanics, and transport physics. Finally, we discuss various computational and simulation studies of nanoparticle adhesion, including advanced treatments that compare directly to experimental results. Future work will ideally continue to combine experimental data and advanced computational studies to extend our knowledge of multivalent adhesion, as well as design the most powerful nanoparticle-based agents to treat cancer.

## Introduction

Cancer is one of the leading causes of death, reaching an estimated 10 million worldwide in 2020 [1]. Traditional cancer management can prolong life expectancy for many patients. However, inefficiencies in detection via anatomical imaging, biopsy, and biofluid sampling [2], as well as treatment via surgery, chemotherapy, radiotherapy, and immunotherapy, can negatively impact patient care by costing valuable time and eliciting adverse reactions. These drawbacks of traditional cancer diagnosis and therapy have progressively driven more researchers to seek out alternative new therapeutic approaches that will satisfy critical needs in terms of sensitivity, efficacy, and safety. In recent decades, nanotechnology has emerged as a new and powerful way of advancing diagnosis and treatment of numerous diseases, including cancer [3]. Specifically, anti-tumor research has increasingly utilized nanoparticles (NPs) as carriers due to attractive pharmacokinetic and biodistribution properties, as well as reduced toxicity to the rest of the body. Recent reviews have highlighted various NP strategies aimed at enhancing imaging for diagnosis and drug efficacy for therapy [4, 5]. Compared to alternative clinical contrast agents, NPs tend to exhibit higher sensitivity and/or specificity for abnormalities such as tumors due to selective delivery [6], including preclinical studies that have shown promise for computed tomography, magnetic resonance imaging, ultrasound, and positron emission tomography (see reviews: [7, 8]). However, only a few NP imaging agents have been approved for clinical use [9]. For therapy, there has been an increasingly large number of nanomedicines for cancer that have achieved preclinical success (see review [10]), but only a handful of NP drug systems have been approved for clinical use [11, 12]. Many of these FDA-approved drugs have ultimately disappointed in translational human trials following exciting performance with in vitro and in vivo models [13]. For example, pegylated liposomal doxorubicin, such as Doxil and Caelyx, have yielded statistically significant survival changes with metastatic ovarian cancer, but failed to elicit a survival advantage for HIV-related Kaposi’s sarcoma and metastatic breast cancer [14].

NPs have been designed to reach desired target sites and enable selective cellular uptake with increased efficacy and reduced toxicity. This can be achieved through passive or active targeting strategies. Nanocarriers designed for passive targeting exclusively utilize the enhanced permeability and retention (EPR) effect for delivery [15]. This results from nanocarrier extravasation into the tumor tissue via “leaky” vessels, followed by accumulation due to poor lymphatic drainage. Passive targeting relies upon the assumption that unique tumor vascularization, which describes the amount and homogeneity of vessels in the tumor, is sufficient to deliver NPs uniformly and efficiently [16]. Because NPs are often designed to be taken orally or through injection, they must first traverse through the bloodstream to the tumor site. Numerous factors influence NP residence time in the blood (pharmacokinetics) and delivery to different organs (biodistribution), but generally these factors are better for NPs than small molecules [17, 18]. Furthermore, the shape and size of the particle plays an important role [19]. Of note, passive targeting localizes NPs to the tumor tissue, but cannot further promote uptake by cancer cells [20]. The drawbacks for passive targeting can be mediated by designing NPs that actively target receptors overexpressed on tumor cell surfaces using antibodies or various other ligands including proteins, carbohydrates, nucleic acids, peptides, and small molecules [20, 21]. This is generally referred to as active targeting, which must still take advantage of the EPR effect to first localize the NPs within the tumor before binding to specific cell surface receptors and, in many cases, internalization. In addition to NP shape and size, which are also important for passive targeting, actively targeted NPs can benefit from the phenomenon of multivalency, which can be used to fine-tune adhesion between the NP and tumor cells [15, 16].

**Fig. 1:**
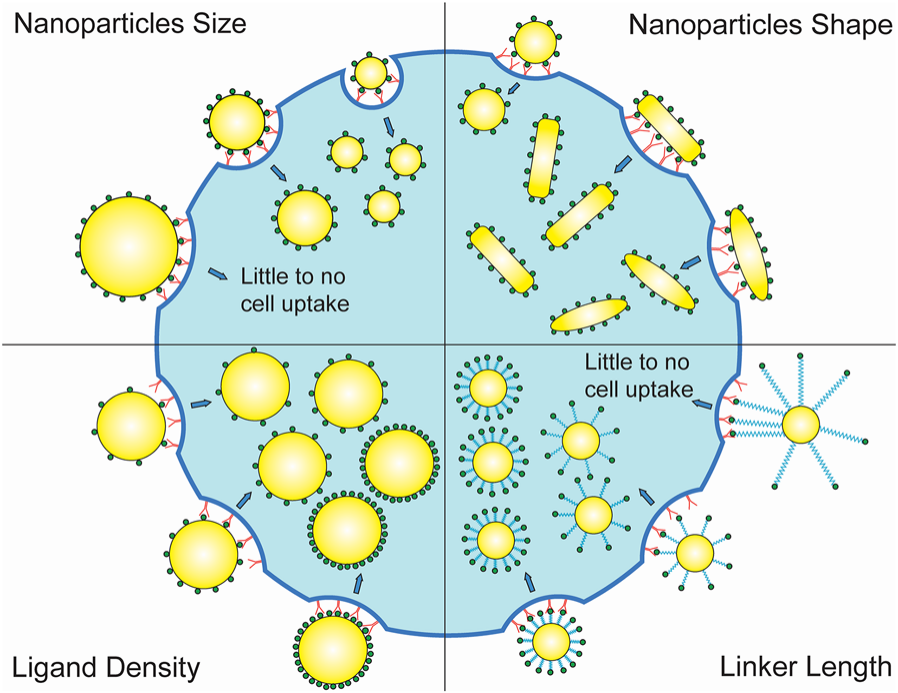
Schematic representation of key parameters influencing NP binding and cellular uptake, including size (top left), shape (top right), ligand density (bottom left) and linker length (bottom right). Yellow represents the NP, green represents ligands, red represents receptors, and blue represents linkers

Interestingly, NPs approved for clinical use for both cancer imaging and treatment have all utilized passive targeting; thus, there is question of whether active targeting holds the potential to improve accumulation at the tumor site and prevent off-target accumulation, primarily within the liver, lung, and kidney. Current active targeting NPs have shown only modest improvement in drug efficacy with little to no contrast enhancement for molecular imaging [22]. Another major challenge has been transitioning in vitro results to in vivo animal models [23], and finally translating to clinical settings. Lastly, appreciation has recently increased for the role of cellular heterogeneity within tumors [24], which further complicates the design and deployment of NPs for cancer diagnosis and/or therapy. Ultimately, the goal is to design NPs with maximal targeting efficacy and selectivity for cancer cells by selecting optimal properties for the NP and targeting ligand (Fig. 1). It may also be advantageous to control whether the NPs are internalized or remain on the surface, depending on site of action for a given drug. Thus, it is critical to evaluate all factors that can influence NP binding and cellular interactions. Since many of the possible factors that influence NP adhesion are difficult to isolate in experimental settings, computational simulations are also utilized to obtain additional insight. These simulations are typically “coarse-grained” to maintain efficiency, which means that they operate at length scales of tens of nanometers to microns and time scales of nanoseconds up to seconds (see review: [25]).

## Cell binding and multivalency

As discussed, active targeting utilizes molecular binding interactions between ligands on the NP and receptors on diseased cells to attain specificity. Studies have demonstrated that ligand-specific targeting enhances NP binding and cellular uptake compared to “naked” counterparts [26, 27]. Furthermore, multivalent systems enable attachment via numerous binding interactions, which improves targeting efficiency relative to monovalent NPs [28], particularly for weak binding interactions [29]. Muro et al*.* observed a dose-dependent increase in bound polymer nanocarriers with higher target receptor surface density both in vitro and in vivo [30]. The increase in binding affinity between the ligand-decorated NPs and the receptors targeted on the cell surface correlated to an increase in surface density of the ligand [31, 32]. However, Wang et al*.* found that high ligand density can induce cell toxicity in Ramos (human lymphoma B) cells after internalization when targeting transferrin receptor [33], suggesting that the connection between binding and cell responses can be complex, and targeting effects may need to be balanced to achieve optimal results.

## Targeting properties

### NP size and shape

Passive targeting via the EPR effect requires that NPs be a certain size to maximize diffusion and extravasation. Studies have also shown that cellular uptake is size-dependent for different cell lines and NP types, with maximal internalization ranging from 30 to 50 nm [34]. Investigation into a citrate-stabilized gold NP interacting with an unilamellar lipid membrane model system showed that gold NPs larger than 50 nm in diameter were not internalized efficiently, while NPs under 10 nm in diameter tended to show collective aggregation on lipid membrane surfaces, forming tubular aggregates with membrane wrapping effects [35]. Jiang et al. further demonstrated that binding affinity increased with NP size up to 70 nm, but concluded that 40–50 nm NPs demonstrated the best internalization for gold and silver NPs coated with Herceptin antibodies [36]. Haun et al. also found an optimized size of 100–150 nm for polystyrene NPs targeting ICAM under fluid flow conditions mimicking a blood vessel [37].

**Fig. 2:**
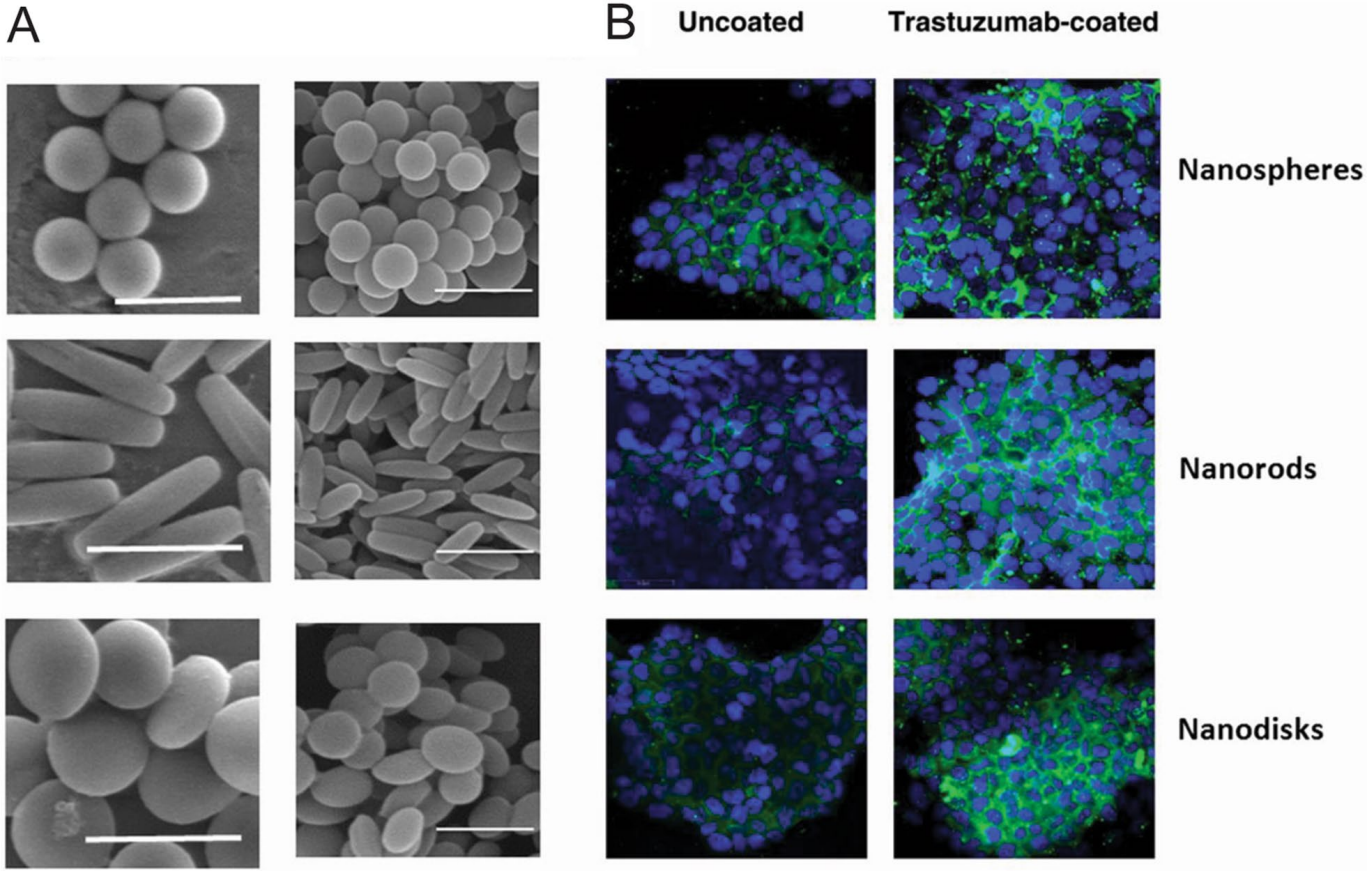
NP shape can affect cellular uptake. **A** Different NP shapes (scale bar: 2 µm left and 500 nm right) **B** Internalization of nanospheres, nanorods, and nanodisks both (left) without and (right) with trastuzumab bound for BT-474 breast cancer cells. Blue represents BT-474 breast cancer cells. Green represents NPs of different shapes. From reference [41]

NPs have traditionally been spherical in nature, but there has been a recent influx in non-spherical shapes (Fig. 2A), including nanodisk, nanorods, elongated liposomes, filamentous polymer micelles/carriers, and carbon nanotubes (see review: [38]). A key general finding without targeting has been that rod-shaped particles collectively have better internalization dynamics than spherical particles for lengths greater than 100 nm [39, 40]. Moreover, longer rods (i.e., higher aspect ratio) facilitated better uptake compared to shorter rods [40]. This observation also held true for targeted NPs, with those larger than 100 nm in length exhibiting better binding and uptake for breast cancer (via trastuzumab), as shown in Fig. 2B, and rat brain endothelial cells (via ovalbumin) [41, 42]. In fact, the difference between nanorods and nanospheres was almost double for the ovalbumin case. Similarly, nanorods over 1 µm in equivalent spherical diameter also displayed better adhesion than nanospheres under shear flow via Sialyl-Lewis A ligands, but the effect diminished for nanorods with an estimated spherical diameter of 500 nm [43]. However, spherical NPs yielded better uptake compared to rod-shaped NPs for sub-micron NPs [44, 45]. In addition, small nano-spheres were also favored over other non-spherical NPs, such as nanodiscs, in binding and internalization via ICAM-1 for endothelial cells [46].

### Ligand density

**Fig. 3:**
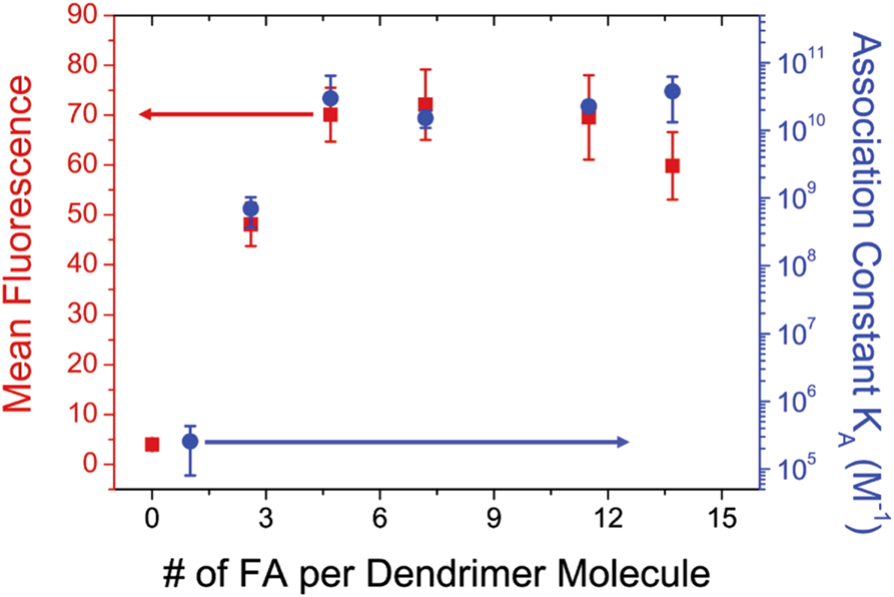
Effect of ligand density on NP binding affinity, showing a plateau above five folic acid molecules (ligands) per dendrimer. From reference [47]

Multivalency is proven to be a powerful strategy for increasing binding and internalization, and hence, ligand density on the NP is a key property. Counterintuitively, however, more ligands is not always better. Hong et.al. used a dendrimer-based NP with folic acid ligands and found that adhesion remained constant beyond ~ 5 ligands per particle (Fig. 3) [47]. In addition, Wang et al. demonstrated that at 25% of the maximum ligand density for both human transferrin (hTf) and transferrin receptor antibody (OKT9), NPs displayed a cellular uptake rate equal to NPs with 100% ligand density [33]. These studies suggest that there may be an upper limit to the binding affinity that can be achieved via multivalency with respect to ligand coating density on the NP, at least in certain contexts and when viewed from the perspective of equilibrium binding behavior.

### Linker length

**Fig. 4:**
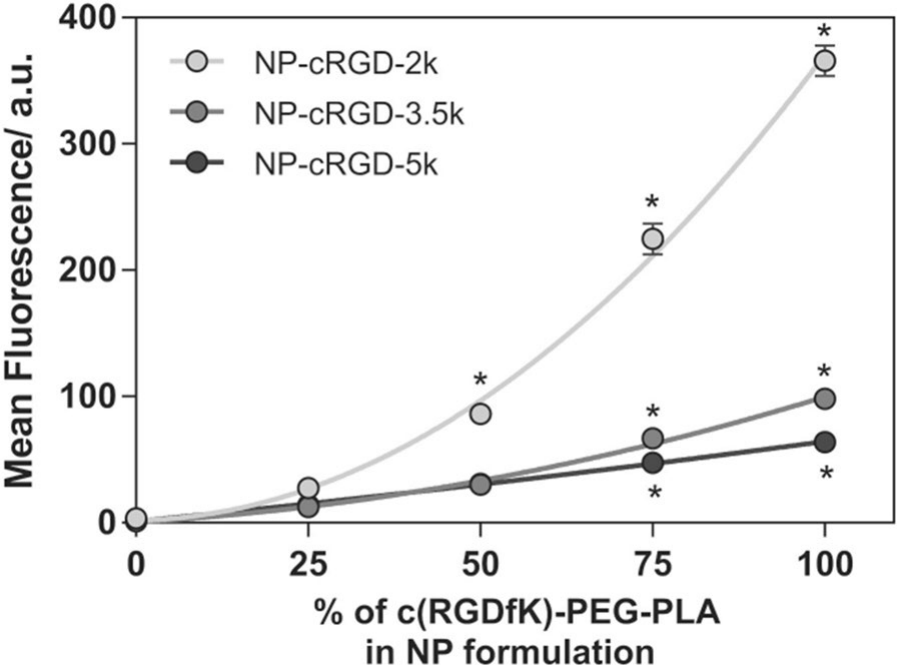
Integrin-mediated uptake of polymeric NPs with different amounts of cyclic RGD bound (0–100%) and PEG linker lengths (2, 3.5, and 5 kDa), showing high density NPs with short linker size result in the best cellular uptake. From reference [50]

Linker length can play an important role by allowing targeting molecules to extend further from the NP surface. Using a recombinant protein system with single-chain antibodies and ~ 200 nm diameter polymer particles, Haun et al*.* demonstrated that insertion of a linker protein fragment improved binding efficiency. However, it was observed that smaller ligand complexes could reach higher coating densities and achieve more efficient binding [48]. Similarly, using double stranded DNA fragments as the linker between the ligand and the magnetic NP, Koets et al*.* observed that binding was better for particles with longer double stranded DNA fragments, in the range of 290 and 560 base pairs, compared to the smallest fragment of 105 base pairs at low density [49]. Interestingly, at higher coating density, there was a shift to the smallest fragment yielding better binding results. This led the researchers to postulate that the enhancement in binding could be attributed to both length and freedom of motion for DNA fragments on the surface, but the freedom of motion was more restricted at higher density. As a result, the smallest fragment had a better binding rate than longer DNA fragments. Furthermore, researchers have shown that both shorter poly(ethylene glycol) (PEG) linkers at maximum ligand density and longer PEG linkers at lower ligand density yield better cellular uptake rates in vitro [50, 51], suggesting that linker length must also be fine-tuned to maximize binding efficacy (Fig. 4). The role of PEG linkers is particularly important, since most targeted nanoparticles used in vitro, and especially in vivo, utilize PEG to reduce non-specific binding.

### Binding properties

As expected, the fundamental binding properties of the ligand-receptor interactions can have a major impact on multivalent NP binding. For example, Csizmar et al*.* designed a NP system with high and low affinity anti-EpCAM fibronectins that were attached at various surface densities. It was observed that reducing ligand density of the high affinity interaction yielded a targeted NP construct that bound well to MCF-7 breast cancer, LNCaP prostate cancer, and SK-OV-3 ovarian cancer cells, but not to MDA-MB-231 triple negative breast cancer cells [52]. Alternatively, the low affinity interaction bound to both MCF-7 and LNCaP cells, but not to SK-OV-3 and MDA-MB-231 cells, at both full and reduced densities. These results suggested that NP avidity can be used to discriminate between different cell types based on expression level. Furthermore, Wiley et al*.* observed that at high ligand density, large amounts of transferrin functionalized NPs accumulated within the endothelial cells that comprised the blood brain barrier, while low ligand density NPs did not bind at all [53]. Interestingly, moderate transferrin density was capable of binding efficiently with transferrin receptors on the luminal side of the endothelial layer, and later detaching on the brain side. This suggests that tuning multivalent binding can lead to better infiltration of NPs at complex and hard-to-reach target sites.

The individual bond kinetic rates, for association and dissociation, have unique impacts that can provide insight beyond the equilibrium affinity [29, 48]. However, these effects of bond kinetics are best characterized by changes in multivalent NP kinetics, which will be discussed in a subsequent section.

## Targeting selectivity

**Fig. 5:**
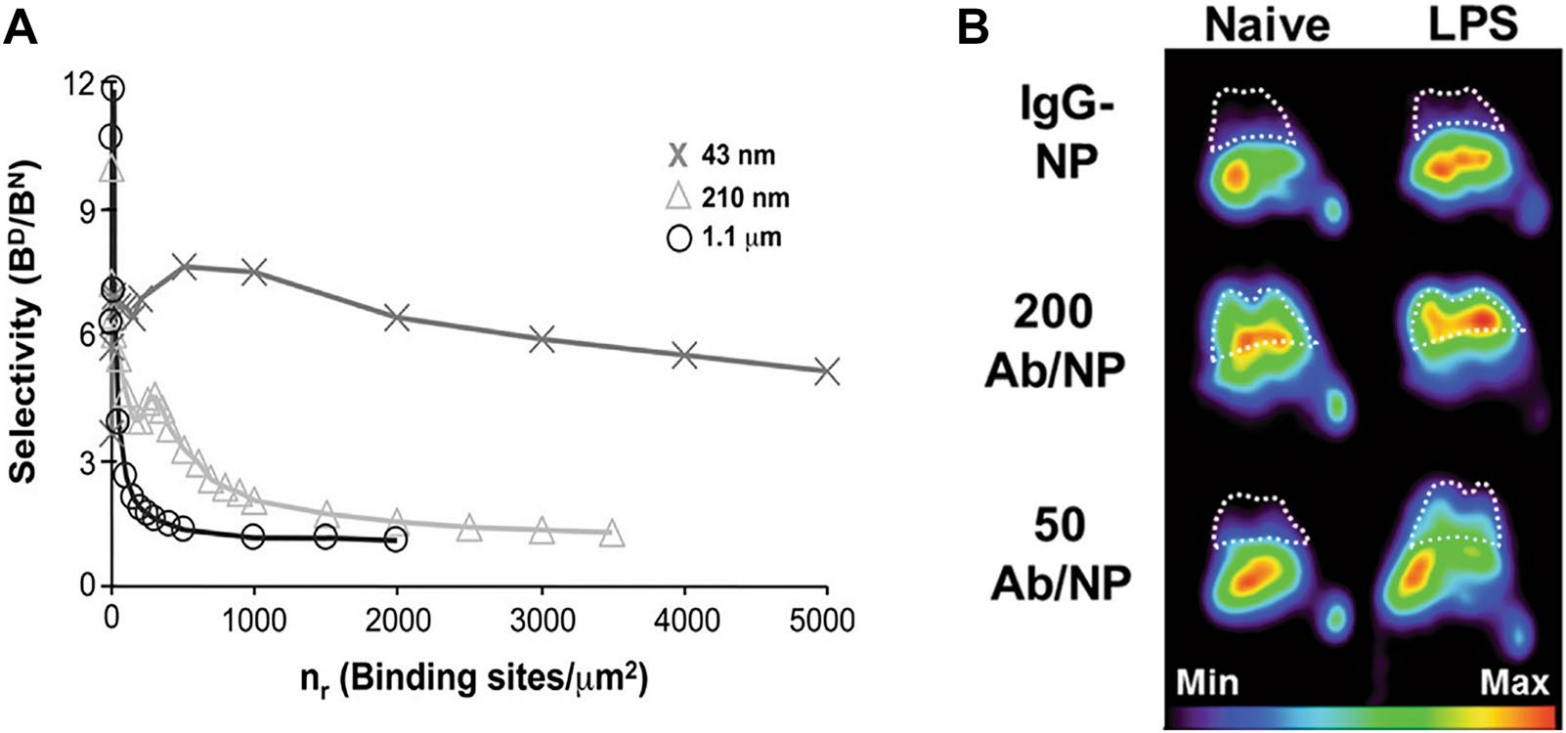
Maximizing NP selectivity in vitro and in vivo. **A** Selectivity, defined as the ratio of NPs delivered to diseased versus normal cells, decreases as receptor density increases for large and moderate-sized particles, but remains high for smaller particles due to elevated diffusion rate. **B** Excessive anti-ICAM-1 ligand density results in high non-specific binding, and thus an optimal signal:noise ratio is obtained at lower density. From references [37] and [56]

An often underappreciated challenge in the targeting field is to discriminate between diseased and healthy cells, which almost universally express the same target, albeit at different levels. Thus, it is important to consider the efficiency of multivalent binding in both contexts. For example, during inflammation there is an upregulation of ICAM-1 expression by endothelial cells from the basal level of ~ 200 sites/*µm*^2^ to ~ 1000 sites/*µm*^2^ [54, 55]. Haun et al*.* predicted from experimental binding data that a 200 nm particle targeted to ICAM-1 would bind nearly as well to a normal cell as inflamed [54]. This is because the binding rate was so high that delivery would be limited primarily by transport limitations. In fact, it was determined that reducing antibody density by half of the maximal level would still retain 90% of the maximum delivery potential. This led to the recommendation that antibody density be decreased to ~ 20% of the maximum value to retain the inherent selectivity dictated by expression level differences. Zern et al*.* later confirmed this recommendation, as they found that reducing ligand density from 200 to 50 anti-ICAM-1 ligands per NP improved the signal:noise ratio in a mouse model of pulmonary disease using a PET imaging platform (Fig. 5B) [56]. Within the context of cancer, several growth factor receptors, such as HER2 and EGFR, are also upregulated on tumor cells compared to healthy cells; thus, it is essential to design a delivery system based on both efficiency and selectivity. As such, tumor cell selectivity via HER2 and transferrin was shown to be optimal at moderate ligand coated densities [57]. Other studies have also shown that NP avidity can be optimized by reducing maximum ligand density, as discussed in review [58].

While it is important to maintain maximum selectivity for diseased cells over normal cells based on expression level differences, it would be advantageous if this went even further. Specifically, there is a long-standing goal to achieve binding level differences that exceed the expression level differential, and even create a switch-like transition in binding between normal and diseased expression levels. This general type of behavior is known as superselectivity and will be addressed later in this article.

## Multivalent kinetics

**Fig. 6:**
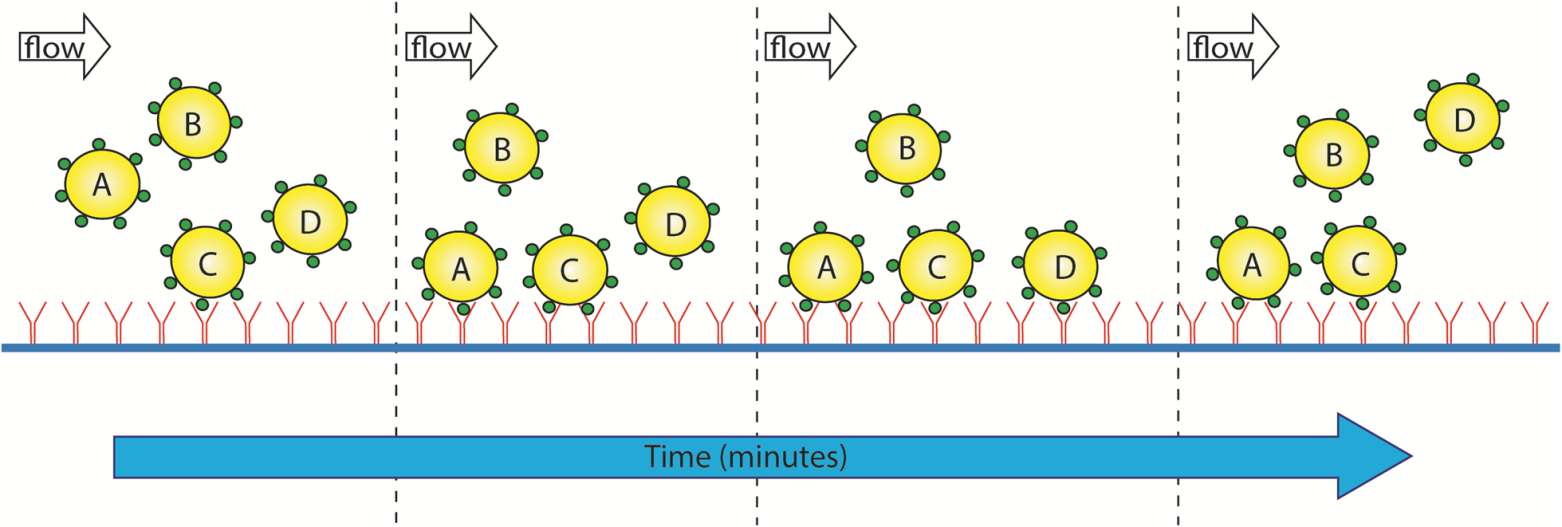
Schematic representation of NP kinetics under flow. Multivalency enhances binding effects

Much of the experimental effort described thus far has been focused on systems that have been allowed sufficient time to reach thermodynamic equilibrium. While this can be informative, assessing NP adhesion from a kinetic perspective can provide additional insight into the processes that govern multivalent binding. For example, Gratton et al*.* found that the rate of internalization corresponded with NP size and shape [40]. Nanorods that were 150 nm in diameter had the fastest rate of internalization, reaching 80% of the maximum value within 25 min before hitting a plateau, indicating a limit in cellular uptake that can potentially be optimized. The rates of initial NP docking, or attachment, as well as the rate of detachment have been assessed using fluid flow-aided experimental setups such as surface plasmon resonance biosensors or imaging in a flow chamber (Fig. 6). Hong et al*.* observed that the attachment rate of a dendrimer NP increased linearly with the number of folic acid ligands, while the detachment rate varied in an exponential manner, indicating that multivalent NP stability on a surface is a complex process [47].

**Fig. 7:**
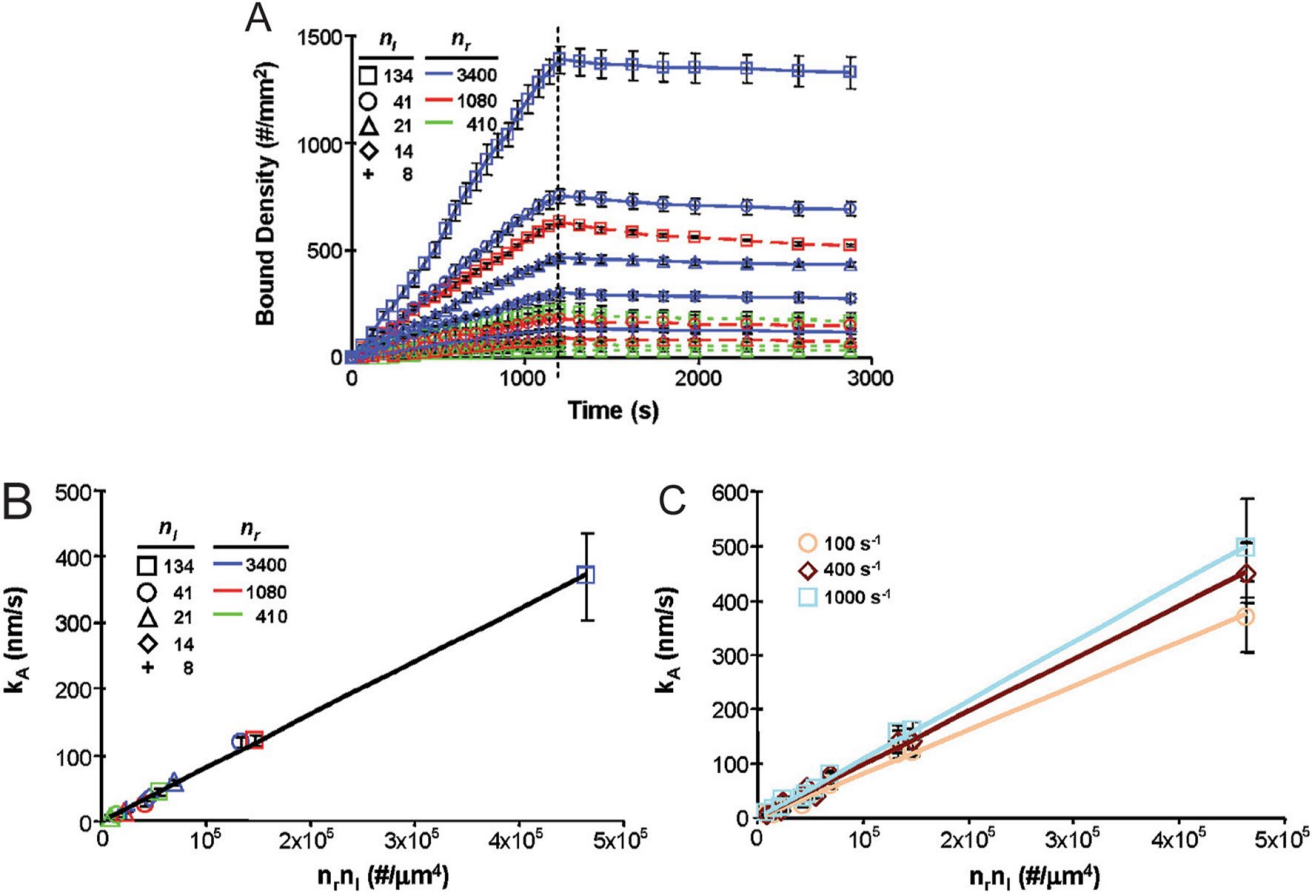
**A** Representation of binding data using ICAM antibody-bound NPs in a flow chamber of varying receptor and ligand densities. **B** Attachment rate constant increases linearly as ligand-receptor numbers increases and **C** for different shear rates. From reference [54]

Haun et al*.* studied the kinetics of adhesion for 200 nm diameter polymer particles mediated by an antibody specific for the inflammatory molecule ICAM-1, and found that NP attachment rate scaled linearly with both receptor and ligand densities (Fig. 7) [54]. Subsequent work demonstrated that the same molecular scaling held for attachment rate using 43 nm and 1.1 µm diameter particles, along with a strong influence for particle size [37]. Specifically, larger particles had a higher intrinsic attachment rate, but the actual level of attachment observed was limited by particle depletion effects, which could be overcome in part by smaller particles due to higher diffusion rate (Fig. 5A).

**Fig. 8:**
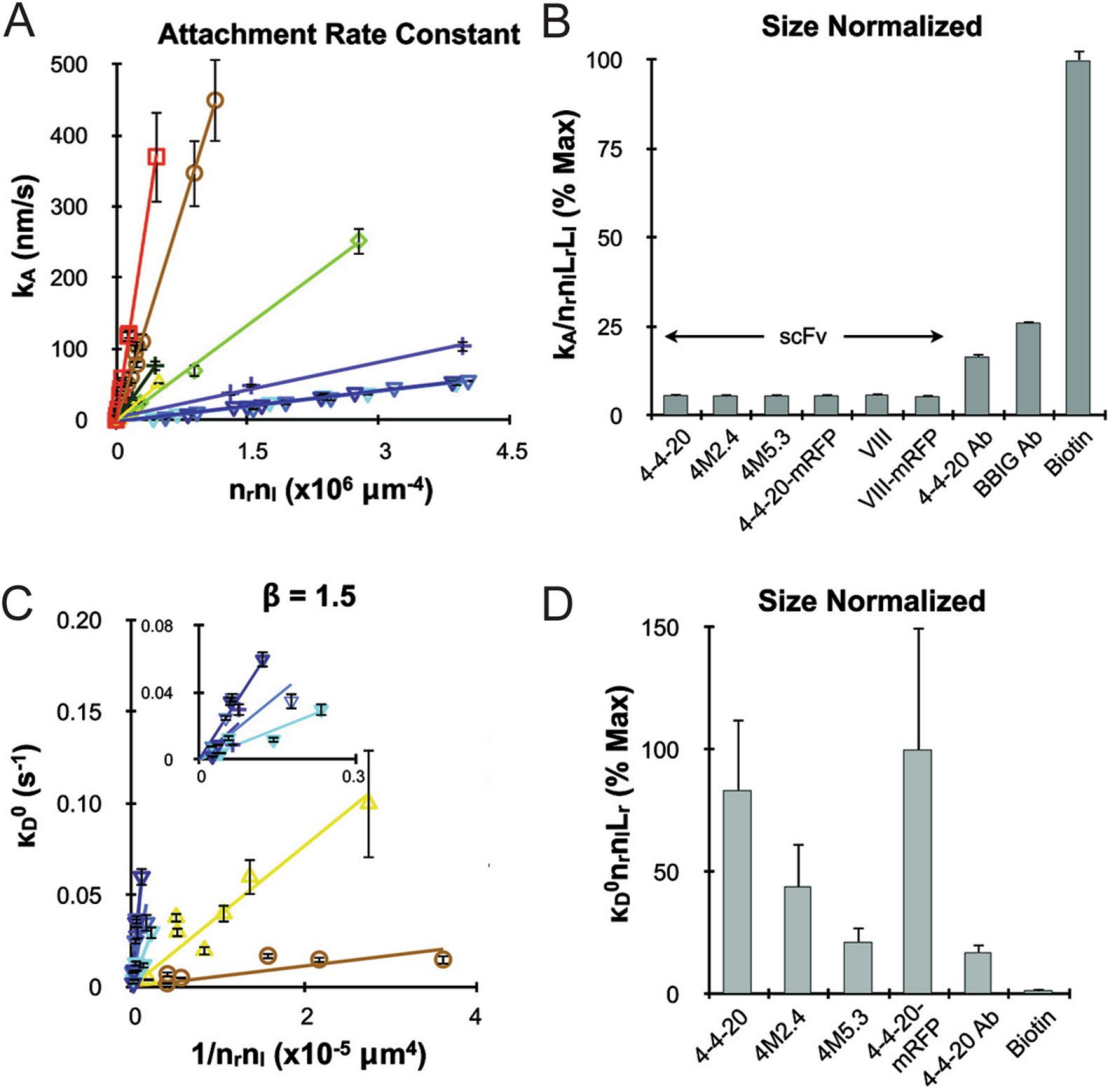
**A** NP attachment rate and **C** detachment rate using single chain antibodies normalized by size (**B** and **D**, respectively). From reference [48]

Interestingly, NP detachment rate was found to be time-dependent, following a power law, acting over a time-scale that spanned the entirety of > 30 min experiments [54], and was remarkably similar for different particle sizes [37]. It was hypothesized that this time-dependency was related to an approach to steady state from an initial smaller number of bonds, followed by additional bond formation that stabilized adhesion, but this could not be verified experimentally. Follow up studies were then performed using a system of recombinant single-chain antibodies, some of which had been evolved to display differences of four orders of magnitude in bond dissociation rate, as shown in Fig. 8A [48]. Surprisingly, NP attachment rate was similar across this single-chain antibody mutant library (Fig. 8B), leading to a hypothesis that initial docking may have been influenced by bond mechanical strength. Specifically, the mechanism would require that one single-chain antibody would be too weak to tether NPs, thus necessitating that multiple bonds form simultaneously. As for the detachment rate, differences between the kinetic mutants were observed following a logarithmic dependence (Fig. 8C and D). Finally, the time-dependent detachment rate phenomena was also observed with single-chain antibodies, but following a different power law.

**Fig. 9:**
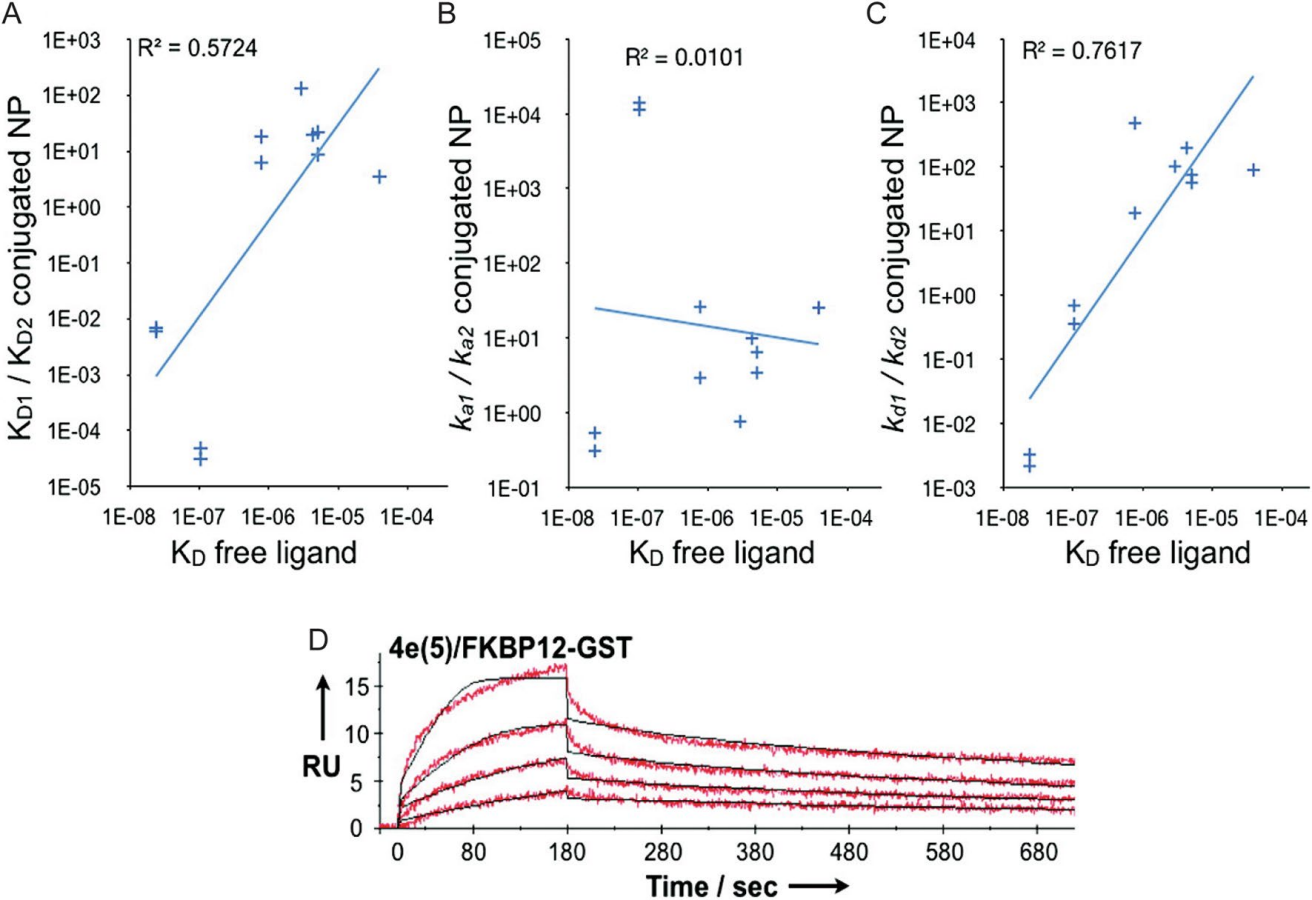
A comparison of bond dissociation constant (K_D_) for free ligands versus the ratio of **A** dissociation constant, **B** association rate, and **C** dissociation rate of bivalent NP. Blue line represents the correlation between free ligands and NP, which is strongest for part **C**. **D** Representation of protein-NP binding data for multivalent NP. From reference [29]

Tassa et al*.* used a system of small molecule (FK506) derivatives attached to cross-linked iron oxide NPs and found a dramatic decrease in detachment rate relative to the dissociation rate of the free ligand and a moderate decrease in dissociation constant as ligand density increased (Fig. 9) [29]. Although not identified by the authors at that time, a clear time-dependence was also evident in the multivalent binding data (Fig. 9D), corroborating that this may indeed be a general phenomenon for multivalent NP adhesion.

## Theoretical and computational modeling of multivalent NP adhesion

The experimental efforts discussed above have provided a strong foundation for our current understanding of NP binding. However, various modeling methods can be employed to bolster this understanding, as well as provide new mechanistic insight. This is particularly true for complex phenomena such as mass transport, mechanical forces, and multi-bond formation dynamics. Furthermore, validated models could be used to explore new parameter space in a manner that is relatively quick and controlled. Purely experimental investigations inherently require large amounts of time and energy, and results are almost universally confounded by differences in numerous variables. Finally, multiscale modeling approaches that consider transport effects, hydrodynamics, bond formation dynamics, and molecular scale interactions will ultimately be required to optimize disparate parameters and produce the best targeted NPs [59], as well as achieve advanced phenomena such as superselectivity. Notably, the predictive power of simulation modeling will improve the likelihood that favorable parameter regimes are discovered and optimized. The following sections will review basic modeling and simulation concepts, starting from basic monovalent bonding through more complex multivalent and NP-specific treatments.

## Molecular bonding

### Monovalent binding kinetics

To fully understand molecular bonding in the NP context, Bell’s work [60, 61] that describes adhesion mediated by reversible molecular bonds between antibodies and antigens is paramount. Allow the classical chemical equilibrium equation to represent receptors on a cell surface (R) and ligands on a NP (L) to be in chemical equilibrium with the resultant receptor-ligand complex (C):

1 $$R + L \rightleftharpoons C$$

For the general case of two complementary receptors, each allowed to move with their own independent degrees of freedom, we can derive the kinetic equation for complex (bond) formation:

2 $$\frac{d\left[ C \right]}{{dt}} = k_{f}^{o} \left[ R \right]\left[ L \right] - k_{r}^{o} \left[ C \right]$$

where [C], [R], and [L] are the concentrations of complex, receptor, and ligand; respectively. The terms $k_{f}^{o}$ and $k_{r}^{o}$ are the intrinsic forward and reverse kinetic rates of complex formation; respectively. At chemical equilibrium, the above rate equation can be set to zero, and simply rearranging the equation yields:

3 $$K_{A} = \frac{\left[ C \right]}{{\left[ R \right]\left[ L \right]}} = \frac{{k_{f}^{o} }}{{k_{r}^{o} }}$$

where $K_{A}$ is the equilibrium association constant, equal to the inverse of the equilibrium dissociation constant, $K_{D}$. This result is known as the Gudberg-Waage, or Mass Action law.

This treatment concretely describes the reaction rates for the receptor-ligand complex from the reaction rates for ligands in solution and diffusion constants of ligands in solution and receptors on membranes. Bell also describes an “encounter complex” which is an intermediate step between the receptors and ligands in solution and the resultant receptor-ligand complex. However, it is a good approximation that the concentration of the encounter complex is small compared to that of the reactants or product, therefore it is accurate to ignore it in the chemical equilibrium equation above.

### Bond biophysics

**Fig. 10:**
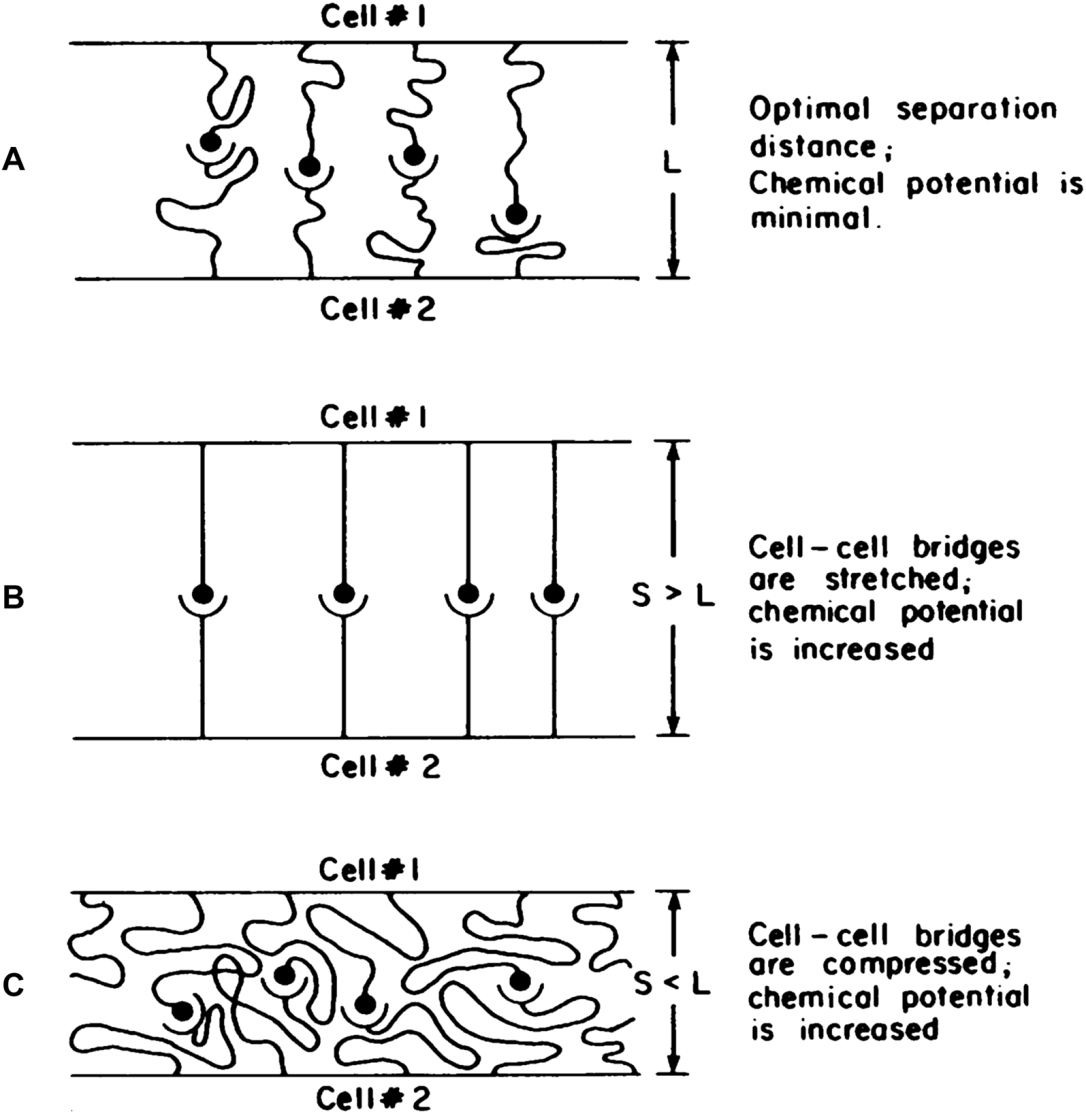
Biomolecular bonds, with average length S, acting as cell–cell bridges. **A** To minimize chemical potential, the length of the individual biomolecular bonds stretch and squish so that the cells are at an optimal separation distance, or equilibrium bond length L. When the biomolecular bonds are **B** stretched with S > L or **C** compressed with S < L, chemical potential will be elevated, which will eventually direct the system back to the equilibrium state (S = L). From reference [61]

Once the reactants and products form a noncovalent bond, the bond can be modeled as a Hookean spring. Under forces that do not lead to rupture, biomolecular bonds will reach a new equilibrium state by minimizing chemical potential, such that the separation distance equals the new bond length, as shown in Fig. 10. Using a Taylor expansion, the chemical potential ($\mu_{b}^{o}$) as a function of separation distance [$S$] can be expressed as:

4 $$\mu_{b}^{o} \left( S \right) = \mu_{b}^{o} \left( L \right) + {\raise0.7ex\hbox{$1$} \!\mathord{\left/ {\vphantom {1 2}}\right.\kern-\nulldelimiterspace} \!\lower0.7ex\hbox{$2$}}\kappa (S - L)^{2} + \ldots$$

where [$\kappa$] is the biomolecular bond spring constant and $\mu_{b}^{o} \left( L \right)$ is the chemical potential at equilibrium, [$L$] is the equilibrium bond length, and higher order terms are dropped as they are not required for accurate experimental validation. The bond spring force, $F_{sp}$, can then be expressed as:

5 $$F_{sp} \left( S \right) = - \kappa S$$

Atomic force microscopy (AFM) experiments have verified bond force for the avidin–biotin biomolecular bond [62–65]. Specifically, the force quantum of an individual biotin-avidin was measured to be 160 ± 20 pN [66]. The value of biomolecular spring constants have been estimated at ~ 0.1 pN/m, based on Bell’s original model [60, 61], but experimental validation has remained elusive.

From the kinetic theory of the strength of solids [67], Zhurkov et al*.* explained that the lifetime of a covalent bond in a solid can be modeled by an amplitude of the reciprocal of the natural oscillation frequency of atoms. Part of Bell’s insight was adapting this equation for solids to a non-covalent, multivalent molecular binding context. Let’s now consider the case where a receptor-ligand complex has formed at some interface, say a NP and cell. Let’s also assume that the bond is under a force load, defined as $f_{B}$, that will act to accelerate bond rupture. The observed kinetic reverse reaction rate, $k_{r}$, can be expressed in terms of the intrinsic value, $k^{o}_{r}$, and $f_{B}$, as follows:

6 $$k_{r} \left( {f_{B} } \right) = k_{r}^{o} e^{{\frac{{\gamma f_{B} }}{{k_{B} T}}}}$$

**Fig. 11:**
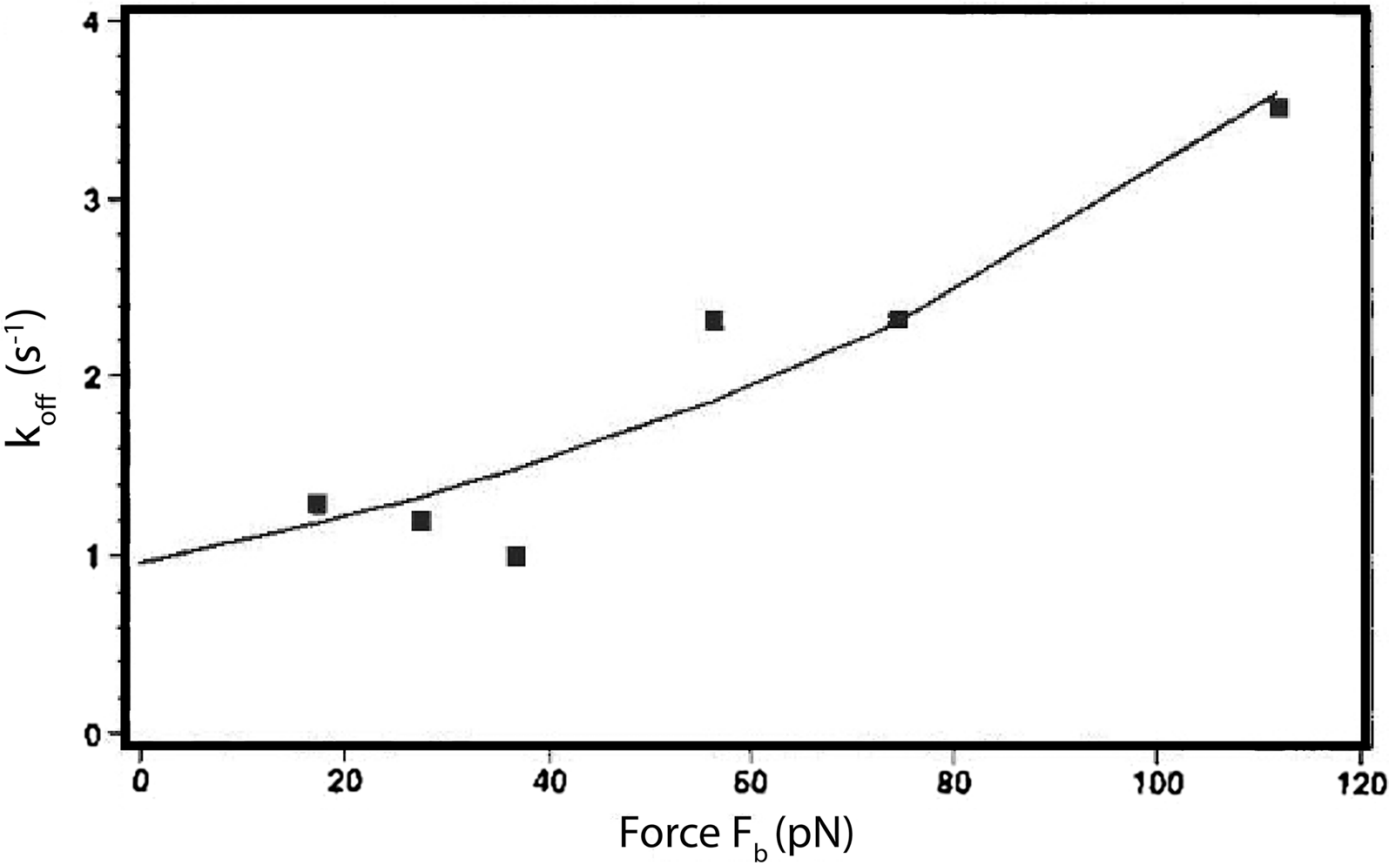
Plot of $k_{r}$ (denoted as $k_{off}$) as a function of bond force $f_{B }$(bond force) for a P-selectin carbohydrate bond with a $\chi^{2} = .484$. This confirms that Bell’s use of Zhurkov’s covalent bond model on biomolecular bonds provides an accurate prediction for the observed reverse reaction rate under force loads. From reference [63]

The difference between this equation compared to Zhurkov’s is that $\gamma$ is now the empirically measured reactive compliance of the bond, in units of length. Therefore, the term $\gamma f_{B}$ dictates the amount of mechanical work that is performed on the bond to drive rupture. We can also interpret $k_{r}^{o}$, from the context of Zhurkov, as the reciprocal of the natural oscillation frequency of a harmonic oscillator. Merkel et al*.* performed single bond rupture experiments using AFM and the avidin–biotin interaction to validate Eq. 6 and quantify the reactive compliance. It was found that bond lifetime varied from 1 min to 0.001 s when subjected to forces ranging from 5 to 170 pN [62]. Moreover, Alon et al*.* [63] plotted the bond rupture force of P-selectin carbohydrate as a function of the force loading rate, and also found agreement with Eq. 6 (Fig. 11). Relaxation time describes the time required to overcome the activation energy to break a bond and minimize the free energy, which follows a harmonic oscillator.

## Multivalent NP adhesion kinetics

### Attachment and detachment rates

For a NP to bind, it must arrive at a vacant site on the target surface. Turitto calculated a flux theory that assessed the probability of a particle reaching an unoccupied site and subsequently binding as the product of the former and the latter [68]. However, in the low-binding density limit, valid either at early stages or low binding efficiency, we can consider only the binding effects. Using an analogy to Eq. 2, but now for multivalent NPs, Haun et al. [54] used the following rate equation:

7 $$\frac{d\left[ B \right]}{{dt}} = k_{A} \left[ {C_{W} } \right] - k_{D} \left[ B \right]$$

where *B* is the bound NP density (number/area), $C_{W}$ is the unbound NP concentration at the wall, *t* is the time, $k_{A}$ is the NP attachment rate constant, and $k_{D}$ is the NP detachment rate constant. $k_{A}$ and $k_{D}$ refer to multivalent reaction rates, which differ from the monovalent reaction rates for the receptor-ligand pair, $k_{f}^{o}$ and $k_{r}^{o}$. It is assumed that the multivalent and monovalent reaction rates are correlated, however, at this time, these relationships have not been established. Haun et al. did demonstrate that $k_{A}$ scaled proportionally with receptor and ligand coating densities regardless of particle size [37, 54], as well as receptor length [48]. The scaling laws for $k_{D}$ were considerably more complex, however, which may have been due to indirect contextual factors that ultimately affected bond numbers. In the limit of low values for *B*, and if the total number of bound NPs (*B*^*Total*^) is known, then a linear attachment rate is obtained by setting $k_{D}$ = 0 in Eq. 7 and integrating:

8 $$B^{Total} = k_{A} C_{W} t$$

Thus, $k_{A} C_{W}$ is given by the slope of $B^{Total}$ vs time. At high reaction rates, NPs can be depleted from the bulk solution so fast that $C_{W}$ decreases, which will appear to represent a lower value for $k_{A}$. These conditions can be anticipated by calculating the Damkohler number, and $k_{A}$ can be isolated from $C_{W}$ using appropriate transport-reaction modeling [54].

Whether a NP attaches will depend on formation of the first bond tether, which can be determined based on the observed rate ($k_{f}$) and probability of bond formation ($P_{f}$). These phenomena have been studied in simulations by describing NP fluctuations between bound and unbound states as thermally-driven jumps over a potential energy barrier. This can be conceptualized as taking place over two discrete steps. First, the NP comes sufficiently close to the surface of interest. Second, bond formation can occur at the intrinsic rate $k_{f}^\circ$. For the first step, a Hookean spring is used to model extension of the unbound molecules towards each other, resulting in the observed reaction rate $k_{f}$:

9 $$k_{f} = k_{f}^{o} exp[\frac{{ - \sigma_{ts} (S - L)^{2} }}{{2k_{B} T}}]$$

where $\sigma_{ts}$ is the transition state spring constant that confers an entropic penalty based on the orientation of adhesion molecules. The probability of formation can then be calculated directly as follows:

10 $$P_{f} = 1 - e^{{{-}k_{f} \Delta t}}$$

where $\Delta {\text{t}}$ is the observed time, or time step in a simulation context. During each time-step, *P*_*f*_ is calculated for each potential bond in a given system and compared to a randomly-generated number. If the random number is less than *P*_*f*_, a bond is considered to have formed. Using this simulation approach, it was found that the maximum extension or compression length for biomolecular bonds is ~ 0.9 nm [61].

### Time-dependent detachment rate

Multivalent NP stability after binding was also found to be complex from a temporal perspective [37, 48, 54]. Specifically, $k_{D}$ appeared to decrease over time, which was referred to as adhesion strengthening. Although the mechanism underlying this effect was unknow, the time-dependency was successfully captured by a phenomenological power law:

11 $$k_{D} \left( t \right) = \frac{{k_{D}^{o} }}{{(t/t_{ref} )^{\alpha } }}$$

where $k_{D}^{o}$ sets the initial magnitude of the detachment rate, α sets the type of time dependent polynomial function, and *t*_*ref*_ is a reference time that was included for unit consistency (value = 1 s).

In a detachment experiment, where buffer is flowed over pre-bound NPs, the constants $k_{D}^{o}$ and α can be found directly from experimental data by substituting Eq. 9 into Eq. 7 and setting $k_{A}$ = 0, arriving at:

12 $$B = B_{o} exp\left[ {\frac{{k_{D}^{o} }}{1 - \alpha }\left( {t_{o}^{1 - \alpha } - t^{1 - \alpha } } \right)} \right]$$

where $B_{o}$ and $t_{o}$ are the initial bound particle density and time, respectively. It is important to note that α represents the power law value that correlates to real-world experimental time. Empirically-determined values for α were found to be ~ 2/3 for different receptor/ligand densities and NP sizes [37, 48, 54].

Since different NPs bound at different experimental times, Eqs. 11 and 12, as well as α, hold little mechanistic value. Therefore, analogous equations were developed in which experimental time was replaced by the bound particle time, *t*_*b*_. To distinguish the experimental and individual NP contexts, $k_{D}$, $k_{D}^{o}$, and α were replaced in Eqs. 9 and 10 by $\kappa_{D}$, $\kappa_{D}^{o}$, and $\beta ,$ respectively. NP bound time *t*_*B*_ can be measured experimentally, but is easiest to track using a simulation, and a simple method was developed by Haun et al. [54]. A detachment probability, *P*_*D*_, was calculated from the time-dependent rate $\kappa_{D}$, using an analogy to Eq. 9:

13 $$P_{D} = 1 - e^{{{-}\kappa_{D} \Delta t}} = 1 - exp\left[ {\frac{{ - \kappa_{D}^{o} }}{{(\frac{{t_{B} }}{{t_{ref} }})^{\beta } }}\Delta t} \right]$$

where $\Delta {\text{t}}$ is again the simulation time-step. Simulations were conducted by adding a constant number of particles to the system during each time step, $\Delta {\text{B}}$, based on the experimentally-measured attachment rate. During each time-step, *P*_*D*_ was calculated for each NP in the system and compared to a randomly-generated number. If *P*_*D*_ was less than the random number, the particle detached, otherwise the bound time was updated for the next time step. This simulation approach determined that the NP-centric power law constant, $\beta$, was ~ 3/4 for most systems studied [37, 48, 54]. The agreement between experimentally determined values of α and simulation determined values of $\beta$ is strong evidence for the predictive capabilities of the Monte Carlo generated time-dependent detachment rate.

## Model considerations for simulating multivalent NP adhesion

### Minimizing thermodynamic free energy

**Fig. 12:**
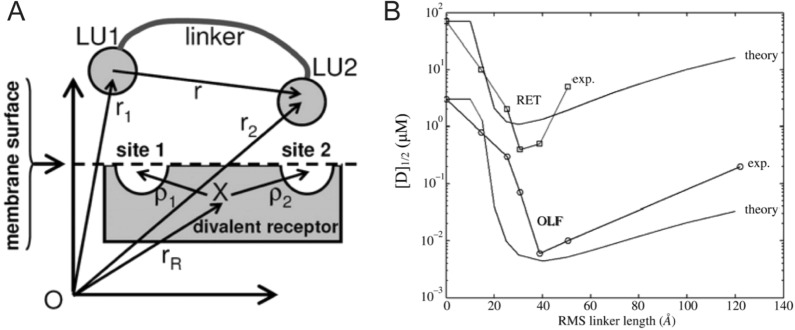
**A** Two ligating units (LU1 and LU2) that comprise component D can bind to receptor R at sites 1 and 2. **B** Graph of [D]1/2, or free receptors, vs root mean squared (RMS) linker length (r) between the two ligating units. An asymmetry in the curve is observed, with a minima around 40 Å, followed by a subsequent slow increase with RMS linker length. Below 40 Å, decreasing RMS linker length causes [D]1/2 to increase rapidly. This confirms that a linker length larger than the minima of 40 Å would tend to reduce the concentration of free receptors, increasing the concentration of bound ligating units, increasing avidity. From reference [70]

An influential work by Wang and Landau presented a Monte Carlo algorithm that simulated thermodynamic and entropic models for calculating free energy, entropy, and phase transformations speedier and more accurately [69]. Diestler and Knapp [70] based their Monte Carlo algorithm on Wang and Landau’s model and used it to model the simplest multivalent binding scenario, a divalent ligand binding to a divalent receptor. This interaction can be understood through Eq. 1, which describes reversible biomolecular bonding. Figure 12A shows two ligating units, LU1 and LU2, are connected by a linker, with length r. The divalent receptor was comprised of sites 1 and 2, which combined on the membrane surface with the ligating units to form the bound complex, D.

Using these initial conditions, along with a chemical potential description of thermodynamic equilibrium, the Hamiltonian, and the Helmholtz free energy, an “enhancement factor” was described that was based on the effective concentration ($C_{eff}$) of ligating units. The enhancement factor predicted that the most stable configuration of a divalent ligand is one where the linker length is the exact same as the distance between the receptor sites. Furthermore, it was shown that if the linker length did not match the separation distance, a longer linker length is preferable. Kramer and Karpen [71] performed a validation experiment on vertebrate photoreceptors (RET) and olfactory neurons (OLF), which are both ion channels that are activated using divalent receptors. The activation of OLF and RET ion channels is shown in Fig. 12B after ion current normalization. The root mean squared (RMS) linker length values at the lowest points on the RET and OLF theory curves are predicted to be optimal based on the enhancement effect predictions by Diestler and Knapp. The predicted values are within a few angstroms of the experimentally calculated values, which shows the accuracy of minimizing thermodynamic free energy with respect to linker length, at least for the simplest case of divalent ligands.

Wang et al. analyzed multivalent NP binding affinity by varying binding energy, ligand functionalization, bond length, ligand density, and NP radius. Key findings were that increasing tether length without increasing ligand density decreased binding efficiency due to tether extension and a conformational entropy penalty from compression. Simultaneously, however, increasing tether length or NP radius increased NP affinity. These predictions were based on Monte Carlo simulations that calculated a probability of bonds forming from conformational entropy of tether extension/compression and thermodynamic free energy models. They showed linear increases in binding efficiency with increases in ligand density for the monovalent particle simulations. The researchers noted that, in order to minimize thermodynamic free energy, the tether length had to be at the equilibrium separation distance [72], similar to Diestler and Knapp.

Kitov and Bundle created a partition function for NPs based on the number of states in a system, which showed an increase in binding strength with multivalency. These favorable bound states result from an increase in degeneracy, through increased in entropy and reduced thermodynamic free energy. The multivalent binding strength of NPs can be improved by rearranging the receptors and ligands to increase the number of possible bonds that can form. This reduced the overall free energy, termed in this context as reducing the avidity entropy. Avidity entropy is a function of the degeneracy of bound states calculated based on the positions of the surface ligands/receptors and the permutations of wall and NP surface curvatures [73].

### Minimizing conformational entropy

**Fig. 13:**
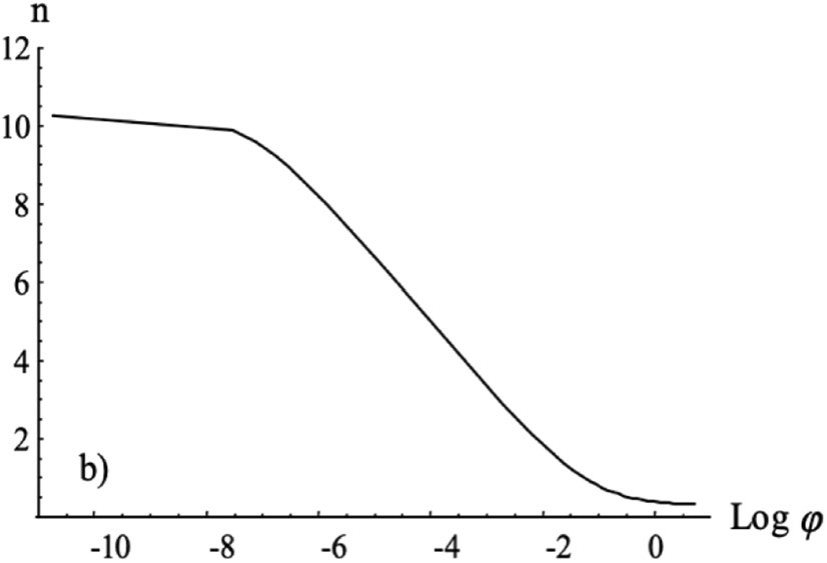
Average number of available bonds per NP (n) as a function of the log volume fraction of colloids. At low NP concentrations, a linear decrease in available bonds per NP is observed, while at larger concentrations the decrease follows a power law. From reference [74]

Researchers simulated a fixed number of NPs diffusing to a flat surface with a fixed density of receptors. By calculating the conformational entropy of the ligand receptor interaction, the receptors on the surface, and the ligated NPs, Fig. 13 shows a linear regime at low concentrations and a power law at high concentration. It is interesting to note that Fig. 13 is an attachment rate that mimics a surface depletion effect, or a reduction in NP attachment due to a lack of available receptors. This result qualitatively confirmed, by experiment, the researchers’ calculations and simulations of minimization of conformational entropy. The two regimes are maintained qualitatively for a small number of receptors [74].

A minimization of conformational entropy host–guest binding for multivalent guests with monovalent hosts was developed that used a similar effective concentration, $C_{eff}$, along with calculations of rate constants and diffusion-limited association. It was found that multivalent interactions improved binding efficiency. Huskens et al*.* showed the linear attachment rate at low concentrations, as well as a power law for attachment rate at high concentrations. The researchers here noted Langmuir-type adsorption behavior. Their results only deviated at high NP concentrations because they could not account for biomolecular bond stability using the Langmuir model. The Huskens et al*.* thermodynamic work on effective concentration and guest host interactions had excellent model fitting with experimental data and was able to determine intrinsic binding constants from only basic assumptions about molecular stoichiometry, geometry, and available data on monovalent interactions [75].

### Molecular motion

**Fig. 14:**
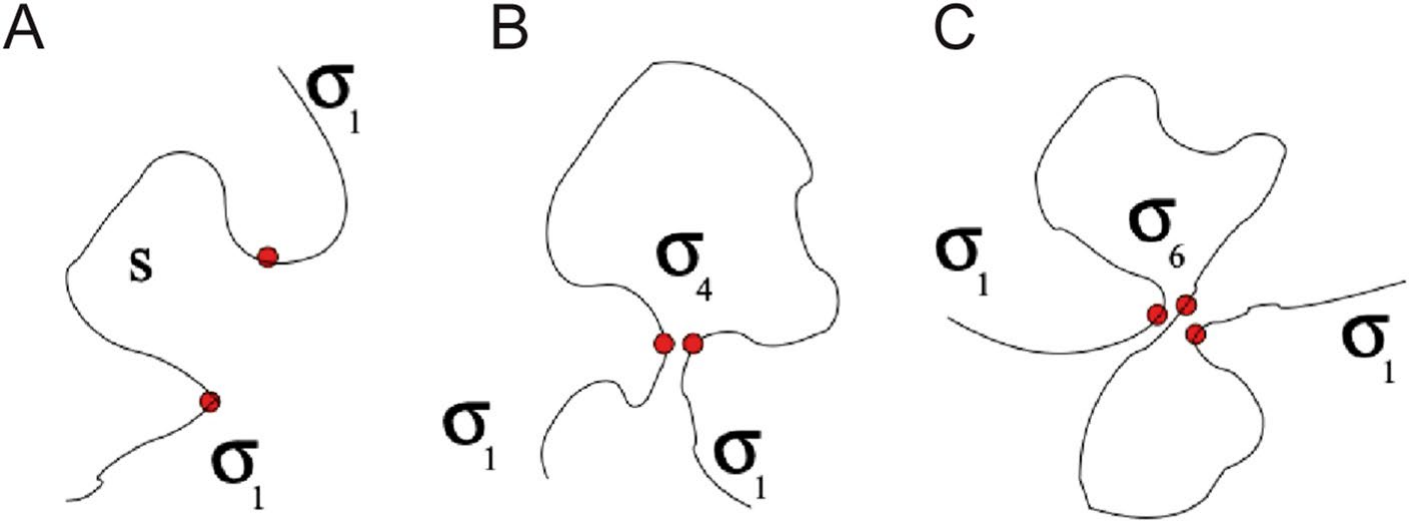
The partition function for polymer position (*Z*) calculated from the number of positions a polymer can occupy for a given length of polymer (*s*), number of chain ends (*N* = 2), and types of vertices ($\sigma_{p}$). $\sigma_{p}$ is proportional to the number of ways the segment vertices can flex. **A** No flexible vertices, therefore the partition function is only proportional to the number of chain ends to the power of the chain end vertex constant ($\sigma_{1}$), or $Z \sim N^{{2\sigma_{1} }}$. **B** There are 2 chain ends and a vertex with 4 possible configurations, or $Z \sim N^{{2\sigma_{1} }} s^{{ \sigma_{4} + 2\sigma_{1} }}$. **C** There are 2 chain ends and a vertex with 6 possible configurations, or $Z \sim N^{{2\sigma_{1} }} s^{{ \sigma_{6} + 2\sigma_{1} }}$. From reference [81]

A Monte Carlo-based kinetic model that described a framework for simulating polymer motion was first created by Deutsch and Binder [76]. This consisted of a bond fluctuation model (BFM) combined with a lattice model for dense polymer solutions and polymer mixtures in three dimensions. The next advance for polymer chains was the Reptation model, which described anisotropic “slithering” of a polymer chain with long flexible tethers, similar to a snake. Reptation dynamics were modeled through real world experimental analyses [77] and computational validation [78]. The Rouse model involves a series of beads connected by Hookean springs, and for short flexible tethers, is more accurate. This is because for short polymer chains, minimal bending occurs, which reduces entanglement. The Rouse model is accurate for depicting the minimal entanglement, while the slithering tubes of reptation are more accurate for long polymer chains in an entangled regime. Classical Rouse simulations work by randomly selecting a direction and distance for each monomer unit to move. The probability of motion is higher if the lattice site being moved to is empty, and the resultant bond vector is allowed. If both variables are favorable, then consequent motion ensues, otherwise a new probability is calculated. However, there is a regime between Reptation and Rouse that doesn’t fully describe polymer chain biophysics [79]. Evans [80] also performed similar work explaining the mechanics of bond formation, but with a computational solution that minimized total free energy for bridges between membranes. This model determined the minimum macroscopic tension to separate the membranes and the maximum macroscopic tension for bridge formation. Evans’ computational solution is an extension of Bell’s original model, which further the understanding and predictions of bond formation. Lee et al*.* described geometrical considerations for a partition function of polymer binding. Incorporating the Rouse model, the researchers considered a chain of monomers that must overcome a volume barrier with osmotic pressure acting as boundary conditions. Their results suggested that chain folding is another key mechanism for efficient internal polymer binding, as shown in Fig. 14 [81]. A similar chain configuration geometrical partition function was described by Sung and Park [82]. Sarkar et al*.* explored numerical models of multivalent adhesion using Brownian motion, polymer melt Reptation models, and crosslinking density for deformable NPs. Researchers explored orientation, deformation, and shear stress, and found an accurate stiffness for crosslinkers. Sakar et al*.* used a coarse grain model of NPs because Brownian motion, polymer melt reptations, and crosslinking dominated the deformation of the NPs [83]. Further work on the motion of flexible NPs was performed by Farokhirad et al*.* [84].

### Diffusion of a sphere

Kinetic models trace their roots to the Langevin equations, which dictate that the mean squared displacement of a spherical NP is stochastic, arising from random fluctuations of solvent molecules, which is also known as Brownian motion.

14 $$m\frac{{d\mathop{v}\limits^{\rightharpoonup} }}{dt} = - \lambda \mathop{v}\limits^{\rightharpoonup} + \mathop{\eta }\limits^{\rightharpoonup} \left( t \right)$$

where *m* is the mass, *v* is the velocity, $\lambda$ is the viscosity, and $\mathop{\eta }\limits^{\rightharpoonup} \left( t \right)$ is noise following from a gaussian distribution. Boulbitch et al*.* confirmed experimentally and theoretically that at small ligand concentrations, the adhesion regime is governed by diffusivity, while at high ligand concentrations, the adhesion regime is governed by ligand receptor association [85, 86]. Non-equilibrium conformations have also been explored [87–91], but are outside of the scope of this article.

### Shear force on a sphere

For situations in which NP adhesion occurs under fluid flow, the role of hydrodynamic shear force, $F_{sh}$, can assessed for spherical particles near a wall using the analysis by Goldman, Cox, and Brenner [92]:

15 $$F_{sh} = \frac{{10\pi a^{2} }}{\mu S}$$

where *a* is the NP radius, $\mu$ is the chemical potential, and S is the length of the NP bond. The term $\mu S$ should be ~ 1 Pa, which would therefore set the shear force acting on a 100 nm particle to be ~ 0.5 pN. This is insufficient force to break most single biological bonds, which are typically in excess of 100 pN [60]. Furthermore, AFM measurements have shown that forces greater than 200 pN are required to break immunoglobulin biological bonds [93].

## Integrated models and simulations of multivalent NP adhesion

### Membrane undulation

Model systems have include rigid, static surfaces, such as glass or plastic, but biological membranes are fluid and always in motion. The dilation and movement of surfaces is persistent due to thermal effects, and can be particularly important in environments with shear stresses and bulk flow. At the nanoscale, relative motion between a particle and a surface is not governed by macroscopic forces such as bulk flow [54], but instead dominated by thermal collisions. As a result, a study by Chung and Yu inspected the effect of a moving membrane surface on a bound NP, with considerations for thermal forces acting upon transport of both the membrane and NP [94]. Utilizing coupled Langevin equations, and governing undulations of the membrane surface using the Helfrich Hamiltonian equation in Monge parametrization, the authors modeled dynamics within a Fourier space under deterministic and stochastic conditions. Inspection of the autocorrelation functions indicated that mobile, fluctuating surfaces reduce the oscillatory behavior of velocity relaxation despite the NP’s obedience to the Maxwell–Boltzmann velocity distribution. Furthermore, the effect of oscillatory modulation increased for a higher $k_{A}$, and lower surface rigidity. In contrast, as surface rigidity increased, it was found that fluctuations in NP position aligned more closely with fluctuations of membrane binding sites, and the distribution of particle-membrane distances became increasingly narrow. Finally, variations in relative NP-membrane position influenced bond strengths; by implementing two locally harmonic stable equilibria, “bond states” were segmented and the transition rates between these bond states were measured. Addition of the moving membrane corresponded to an increase in the rate of bond state transitions [93].

A biophysical model of NP adhesion was developed by Radhakrishnan et al*.*, which computed membrane entropy through two main simulation techniques [95]. First, the researchers separated the oscillating harmonics of a membrane through principal component analysis and Fourier decomposition of Helfrich energies, as well as application of the equipartition theorem. After accounting for the stretch modulus of the membrane, both methods agreed on membrane entropy calculations. Furthermore, these membrane entropies were on the same order of magnitude as the NP conformational entropy and NP-receptor binding enthalpy. The enthalpy-entropy relationship between the formation and destruction of bonds on spherical NPs showed that structural flexibility promoted higher levels of multivalent adhesion and stronger avidity, as compared to rigid spherical NPs [95]. The same group later compared their prediction to other groups using NPs injected into a mouse model [96, 109]. However, the authors were quick to exercise caution when interpreting this agreement between simulation and real-world experiments due to modeling approximations. More research is required to confirm whether shear rate is significant in vivo for flexible NPs, but simulation results are not promising. Simulations have shown that micron-sized particles display better margination independent of shear rate. However, this work failed to consider that micron-sized particles would increase shear force acting on bonds, leading to decreased avidity. Another interesting conclusion from this paper was that although spherical particles have a higher margination effect, ellipsoidal particles display enhanced adhesion due to slower rotation, which is caused by a higher moment of inertia [97]. Based on these conclusions, a hypothetical way to decouple these disparate effects could be to create spherical microparticles with ellipsoidal NPs inside. Once the microparticles reach a critical distance from the endothelium due to margination, the ellipsoidal NPs would be released. This would achieve enhanced margination without sacrificing affinity. A mechanism that would allow microparticles to be aware of their distance from the endothelium is not currently known, but could involve a surface enzyme or soluble factor.

**Fig. 15:**
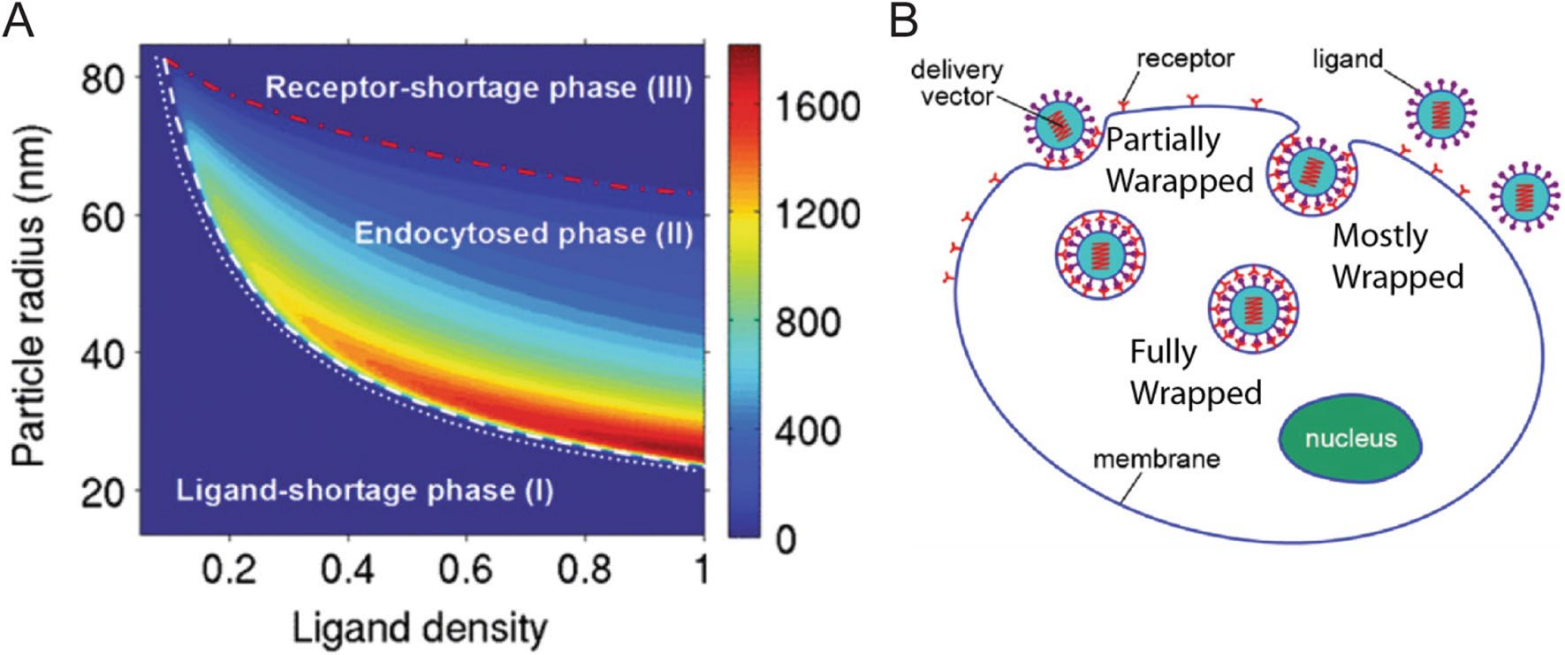
**A** Plot of NP Radius vs Ligand Density, given as a percentage of surface coverage, showing that varying adhesion strengths leads to a phase diagram. Phase (I) describes a lower limit to ligand density compared to the density of receptors, and is known as the “Ligand-Shortage Phase”. Phase (III) is the upper limit to tunable endocytosis when there are too few receptors on the surface as compared to the number of ligands available for binding to the surface, and is known as the “Receptor-Shortage Phase.” Finally Phase (II) describes NPs with matching receptor and ligand densities adherent enough to overcome the limiting enthalpies and membrane undulation entropy, is known as the “Endocytosed Phase”, and is shown between the dotted white and red lines. **B** Schematic showing different levels of wrapping behavior for multivalent NPs. From reference [98]

Yuan et al*.* used simulations to demonstrate that NP radius and ligand/receptor density exhibit a phase transition with exiguous ligands and endocytosis [98], as shown in Fig. 15A. After estimating NP wrapping, or the amount of membrane coating during endocytosis (Fig. 15B), from a Boltzmann distribution, their analyses showed that wrapping is driven by adhesion strength but penalized by membrane deformation. Through limiting conditions of enthalpic contributions, a lower bound of ligand density and particle radius can be found. The lower bound of ligand density is a good predictor of high binding affinity and endocytosis due to its accurate contribution to membrane deformation calculations. Below these lower bounds, they found the “Ligand-Shortage Phase” (I), or that ligand density was too low to support NP binding. Alternatively, there is an upper limit to tunable endocytosis when there are too few receptors on the surface as compared to the number of ligands available for binding to the surface, which is why particle radius is a factor, known as the “Receptor-Shortage Phase” (III). This upper bound on ligand density and NP radius was also set by an entropic limit, above which adhesion was too weak to overcome membrane deformation. NPs with matching receptor and ligand densities are adherent enough to overcome the limiting enthalpies and membrane undulation entropy, this is known as the “Endocytosed Phase” (II). The ratio of high ligand density, ~ 80–100% surface coverage, to small particle radius, ~ 22–25 nm, was the sweet spot for maximum endocytosis, correlated with high attachment rate. However, an asymptotic decrease was observed at a particle radius of 20 nm, which is the limit where decreasing particle radius negatively impacts endocytosis due to an inability to overcome the membrane undulation entropic barrier [98]. Wrapping thermodynamic analyses have also been independently verified by Meng and Li [99].

### Superselective adhesion

There is a great deal of interest in designing environments where NPs bind to regions of high receptor density, while leaving sparse receptor regions largely unaffected [100]. This sharp variation between receptor density and bond formations was discussed in a previous section, and defined as superselectivity. Particularly for most types of cancer, identifying receptors that are entirely unique to the diseased state is a major challenge. Therefore, it is useful to take an approach that selects between diseased and normal cells based on receptor density [101–103]. This could include multivalent binding via a single target receptor, or possibly multiple different receptors, but the ultimate goal is to induce a switch-like transition in binding efficiency for multivalent NP adhesion [104]. Such behavior would be ideal for physiological contexts that demand high contrast and resolution, as well as minimal off-site effects from binding to normal cells. Numerical simulations have already elucidated the fact that monovalent adhesion cannot yield superselectivity, and both numerical simulations and experimental testing have demonstrated that superselective regimes correlate with nonlinear increases in receptor binding arrangements, not by highly varying binding rates [98, 100].

**Fig. 16:**
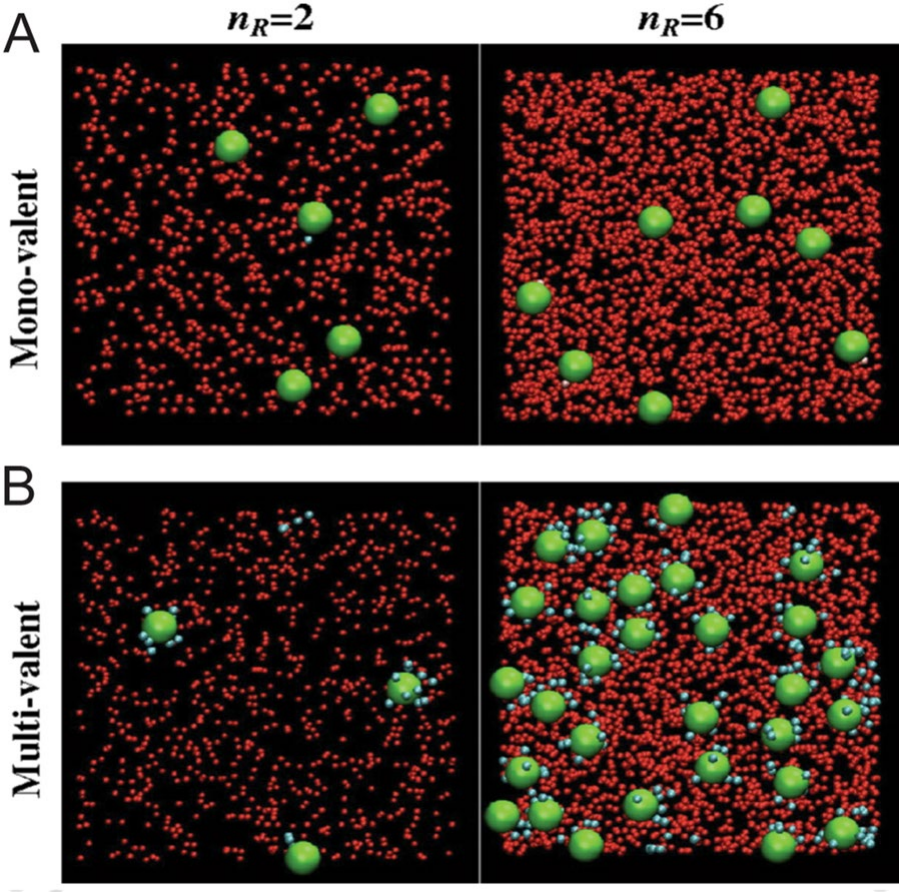
State-based Monte Carlo simulation results prediction superselectivity. Under the monovalent case, increasing receptor densities vary by a factor of three increased binding efficiency by < twofold. With multivalent binding, however, binding efficiency increased by a factor 10. From reference [100]

Multivalent adhesion has been understood by grouping states based on thermodynamic free energy. In a seminal work, Martinez-Veracoechea et al*.* [100] built upon the partition function from Kitov and Bundle [73] to create a grand canonical partition function that described the number of states of a system that can exchange both heat and particles with the environment at a fixed temperature, volume, and chemical potential. This produced the common Langmuir adsorption isotherm (adsorption function), which showed the adsorption probability’s reliance on receptor density. The researchers first demonstrated that monovalent binding behavior could only linearly increase the adsorption function with respect to receptor density. Therefore, researchers submitted two possibilities for superselctive multivalent adhesive behavior. NPs can attach with many short and stiff bonds or a small number of long flexible bonds. In both cases, the coarse-grained Monte Carlo simulations validated the analytical adsorption function with nonlinear growth with respect to receptor density, as shown in Fig. 16.

### Nano adhesive dynamics (NAD) simulations

As discussed, Haun et al. performed a series of flow chamber binding experiments that established relationships between multivalent NP attachment ($k_{A}$) and detachment ($k_{D}$) rate constants and various parameters including receptor/ligand surface densities, flow rate, NP size, and monovalent binding kinetics ($k_{f}^{o}$, $k_{r}^{o}$) [37, 48, 54]. To better understand these relationships, and further study the time-dependent detachment rate phenomenon, Wang M et al*.* developed a simulation method that combined Brownian motion, hydrodynamic forces, and a stochastic treatment of bonding [105]. A Monte Carlo method was used to create distributions of NP dynamics to extract the expected rate value for bond formation and rupture ($k_{f} , k_{r}$; Eqs. 6 and 9), and subsequently the probabilities for bond formation and rupture ($P_{f} , P_{r}$; Eq. 10). The Bell model for binding was used to describe binding kinetics and HIV docking model, called Brownian Adhesive Dynamics (BRAD) [102, 103], was used to model NP motion via a refined Langevin equation. By leveraging these methods to study NP adhesion, the resulting simulations were named Nano Adhesion Dynamics (NAD).

**Fig. 17:**
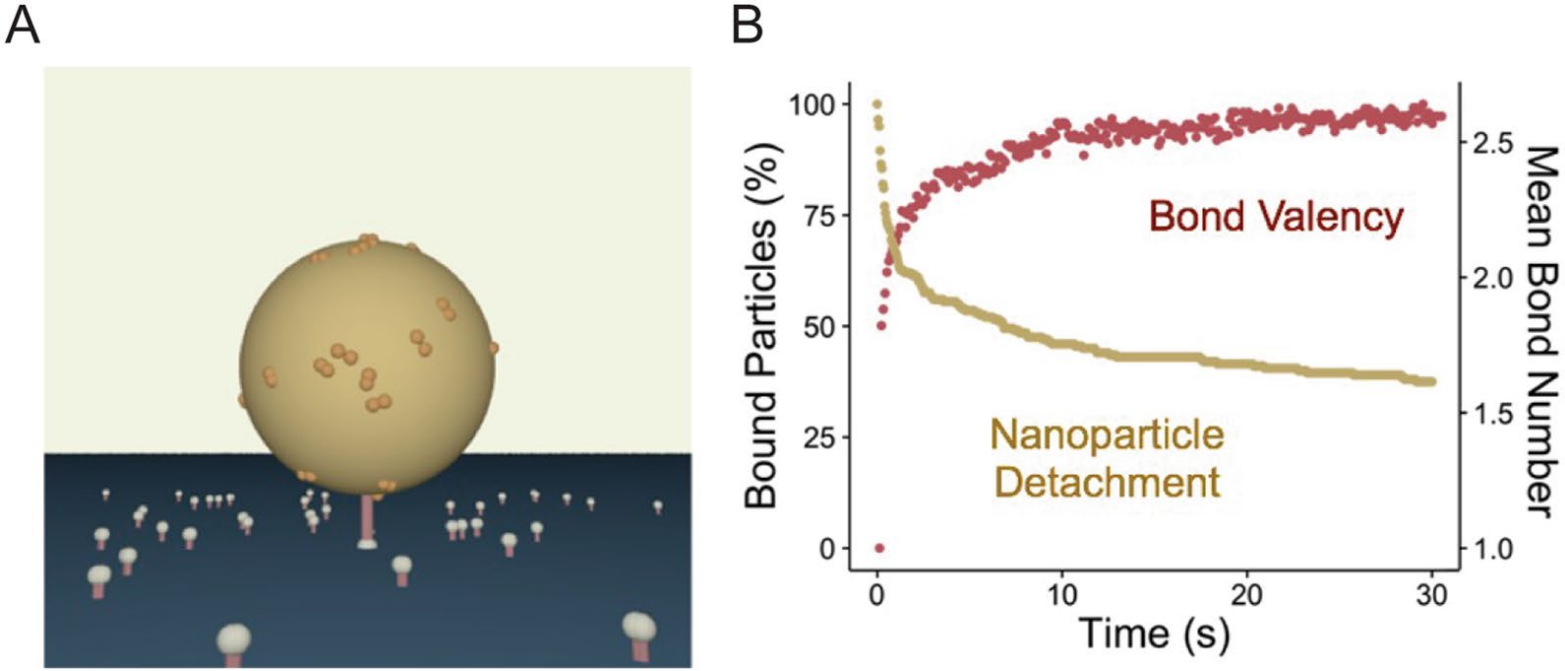
NAD simulations. **A** NP decorated with antibody, flat surface decorated with ICAM-1 molecules, and a single bond tether. **B** Plot containing both NP detachment data and a trace of mean bond number. The detachment curve shows a slowing rate characteristic of the time-dependent phenomena. This corresponds to an increase in mean bond number. From reference [105]

NAD simulations were developed to model experimental data obtained using a 200 nm diameter sphere and adhesion mediated by an antibody to the vascular adhesion molecule ICAM-1 [54]. The focus was only on detachment after NP docking, since this simplified the simulation and was related to the biggest open questions. Simulations were initiated by generating the NP and a flat surface, then decorating the former with antibody and the latter with ICAM-1 at the appropriate molecular sizes and densities. Next, a single bond was formed that tethered the NP to the surface (Fig. 17A). During each time step, the NP was rotated and translated based on the Langevin treatment, bond breakage was assessed using the Monte Carlo algorithm, and unbound ICAM-1 antibodies were sampled for bond formation. By combining the results of multiple detachment simulations into an ensemble detachment profile, the resultant curves could be fit using Eq. 12 to obtain the parameters, *k*_*D*_^*0*^ and $\beta$. Under a large subset of conditions, time-dependent detachment rate behavior was clearly observed. Upon inspection, a correlation was then identified between time-dependent detachment and situations in which bonds were subjected to high mechanical forces. Specifically, these forces were as high as 300 pN, which is sufficient to rupture bonds. Moreover, the source of bond stress was Brownian motion of the NP, which imparted an entropic penalty to the underlying bonds. Another key finding was the apparent time-dependency of detachment rate was caused by heterogeneity in terms of NP binding ability, as some NPs were able to form more bonds than others. Over time, those restricted to lower bond numbers were removed from the system by detachment, which progressively evolved the remaining NP population towards higher bond numbers and overall adhesion stability (Fig. 17B). Based on this insight, it was concluded that it will not be possible to characterize a population of NPs using a single multivalent detachment rate, and for that matter, avidity. Instead, a series of detachment rates co-exist together, each corresponding to sub-populations that possess unique bond numbers and dynamics [105, 106]. This simulation is unique because it correctly predicted the power law detachment of NPs in experiment. As anticipated, the mean bond number monotonically increased during the first second a NP is bound. Over a longer period of time, an increase in mean bond number was attributed to detachment of NPs with low bond valencies due to low bond strength [105].

**Fig. 18:**
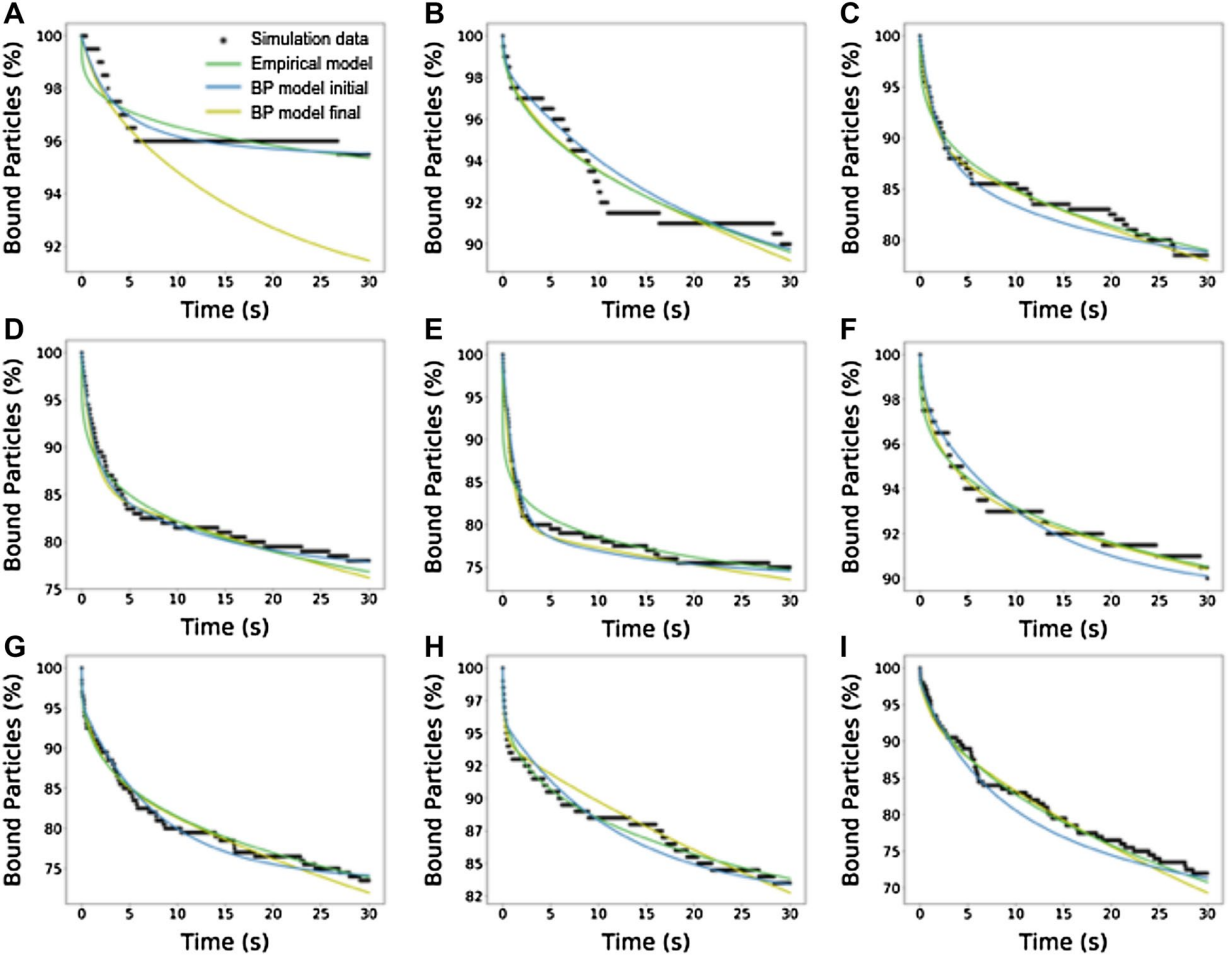
Fitting of time-dependent NP detachment curves using the population-scale detachment model for different experimental conditions including the **A** base case, **B** low antibody density, **C** low ICAM-1 density, **D** ICAM-1 dimers, **E** clustered ICAM1 dimers, **F** $\gamma$ = .29 nm, **G** $\gamma$ = 0.3 nm, **H** $k_{r}$ = $5 \times 10^{ - 4} s^{ - 1}$, **I** $k_{r}^{o}$ = $10^{ - 3} s^{ - 1}$. From reference [107]

Building on the latter observation regarding NP binding heterogeneity, Wang et al*.* developed a population-scale detachment model [107]. The key was to develop the concept of Bond Potential (BP), which was the maximum number of bonds that each NP could achieve due to availability of free receptors or ligands. This BP varied because both molecules were randomly distributed. Using NAD simulation results, a constant detachment rate was assigned to each BP category, and an aggregate detachment rate was calculated for the entire population. The empirical BP model accurately predicted experimental results, as shown in Fig. 18.

### Validation of computational frameworks in vitro and vivo

**Fig. 19:**
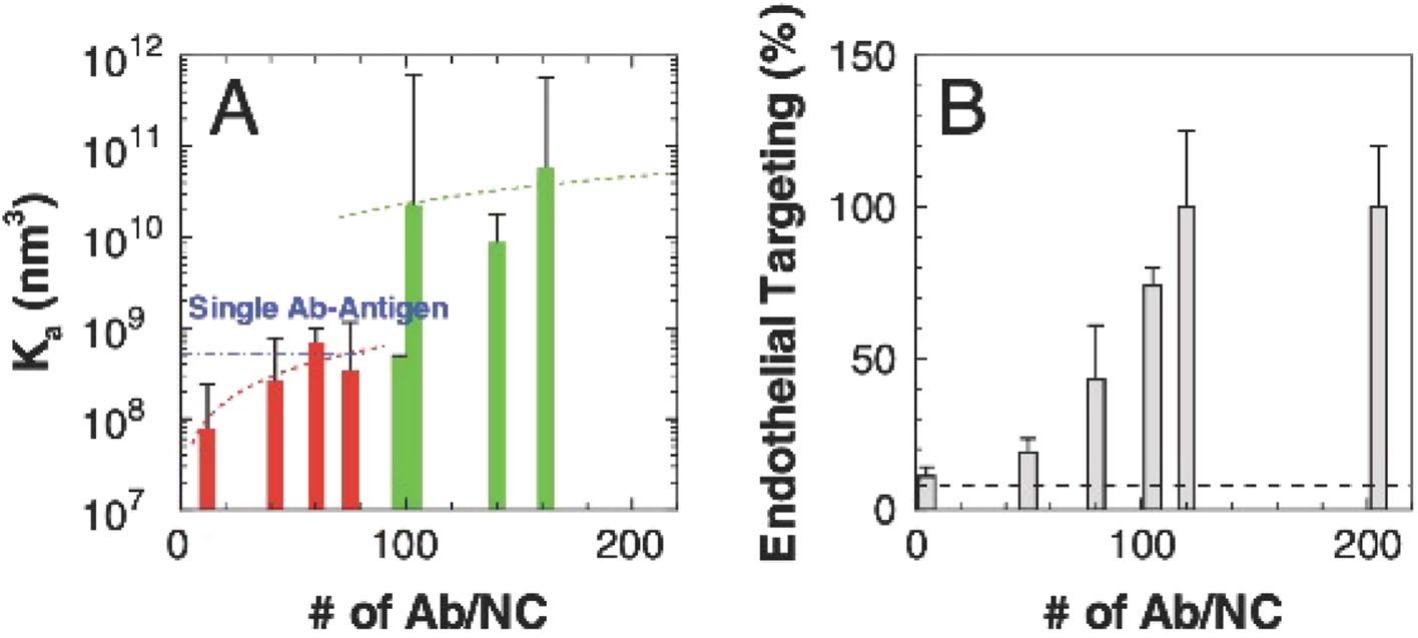
**A** Multivalent NP attachment rate ($k_{A}$) varies as a function of the number of antibodies per nanocarrier, where Ab/NC spans 12–162 (5–74% surface coverage). **B** Endothelial cell targeting percentage vs #Ab/NC. The simulation results from **A** strongly correlates with the endothelial targeting percentage in **B**. From reference [108]

Liu et al*.* developed a Monte Carlo algorithm based on the weighted histogram analysis method (WHAM) that accurately predicted free energy landscapes and quantitatively agreed with in vitro cell binding assays for 50 nm diameter NPs with ICAM-1 [108]. Additionally, the calculated NP free energy space produced binding affinities that agreed with in vitro AFM measurements. NP simulations varied antibody surface coverage ($\sigma_{s}$ defined as antibodies/NP), and a threshold $\sigma_{s}$ appeared at 100, below which the attachment rate drastically reduced (Fig. 19A). Figure 19A is split between a Potential Mean Force calculation of 3 mean bond valencies (green) and 2 mean bond valencies (red). Thus, the researchers demonstrated that a mean bond valency shift of 3 to 2 is significant enough to cause an exponential decay in attachment rate. Moreover, the trend for $\sigma_{s}$ in Fig. 19A concurred with the in vitro vascular endothelium targeting data shown in Fig. 19B, as well as AFM force rupture experiments. This is an excellent example where simulation and modeling enhanced experimental results to provide previously unverifiable insights.

### Additional computational frameworks

The following methods are out of the primary scope of this review, but worth noting as novel targeting approaches. For micron-Sized NPs, Decuzzi et al*.* described three key biophysical steps starting when the NP is within a short range, tens of nanometers, of a ligand receptor. A transient bond is formed, and immediately there is a viscoelastic elongation of the bond, tethering it to the surface. The second step is deceleration of the bond-NP system and a resultant rolling across the surface of the endothelium. The final step is an extension of the bond-NP system across the cell endothelium which constitutes firm adhesion. The bonds created between a NP and a cell wall are balanced between short range attractive forces, Van Der Waals interactions, short range repulsive forces, and steric forces from overlapping electron clouds [109].

Molecular Dynamics (MD) simulations model forces on each individual atom and molecule within a system, moving through short time steps (pico to nano seconds), and provide accurate stochastic equations of motion that can be used to predict the behavior and shape of nano and pico scale objects [110]. Mackenzie et al*.* used MD simulations to provide a molecular-scale view of docking dynamics, which can provide more detailed insight into multivalency, binding free energy, and entropic losses. Specifically, the authors showed that, for NPs smaller than 350 nm in diameter, binding affinity is dictated by a balance of enthalpy and entropy, whereas for larger NPs, the enthalpy of binding is the strongest predictor. Furthermore, anisotropic NPs have a higher propensity for multivalency as compared to their spherical counterparts. Finally, it has been shown that varying ligand type can modify binding affinity without changing multivalency [111].

Physiologically Based Pharmacokinetics (PBPK) models seek to understand how targeted NPs would behave inside the body through a mathematical description of adsorption, distribution, metabolism, and elimination. A key PBPK model was developed by Radhakhrishnan et al*.*, where simulation modeling and real-world experimental results confirmed that higher red blood cell volume fraction increases NP margination, which increased relative binding of flexible and nonflexible NPs. The model indicated roles for protein expression and biomolecular bond mechanics that were validated upon intravenous NP injection into mouse models [96]. The researchers claimed that higher shear rate leads to enhanced targeting of flexible NPs, known as the shear enhancement margination factor. However, this effect was only marginally significant [112]. Furthermore, it has been shown, through agreement between model predictions and tissue experiments in vivo in mice, that shear forces and volume fraction effects from hydrodynamic transport improve functionalized NP disease site targeting efficiency. This effect does not originate from multivalent binding. Furthermore, grouping NPs by bond number has been shown to predict the time-dependent detachment of functionalized NPs from the surface [104].

## Conclusions

Nanomedicine has emerged as a potent technology that holds the promise of being safer and more effective than traditional cancer diagnosis and treatment methods. Although success in clinical settings has remained elusive to date, active targeting holds exciting potential to enhance NP efficacy so that nanomedicine can become part of regular patient care. This is particularly true if multivalent binding interactions can be fully leveraged to maximize accumulation at, and internalization within, tumor cells. However, as discussed in this review, numerous NP design parameters influence multivalent adhesion. Key parameters discussed included, but are not limited to, NP size, NP shape, ligand density, ligand linker length, and receptor density. Additionally, the kinetic rate of multivalent bond formation, from single tethers to the final equilibrium state, provides additional control mechanisms, but also higher complexity. Thus, computational simulations of multivalent NP binding are increasingly needed to track the effects for individual parameters in a controlled manner and explore parameter space in a manner that would never be tractable for experiments. As our understanding of multivalent adhesion and computational power have improved over the years, ever more complicated simulations have been developed that simultaneously account for transport physics, bond stochastics and biophysics, and kinetic treatments of NP adhesion. These works have greatly added to our understanding of avidity, superselectivity, mechanical forces, and novel adhesion phenomena. Most importantly, these computational works are beginning to directly incorporate experimental data that serves as critical validation. Future efforts will ideally be focused on combined experimental and computational studies of multivalent NP adhesion to add to this knowledge base, as well as enable design of the most powerful targeted NP-based diagnostic and therapeutic agents for cancer.

## Data Availability

Not applicable.

## List of symbols

$k_{f}^{o}$: Monovalent forward reaction rate
$k_{r}^{o}$: Monovalent reverse reaction rate
$k_{f}$: Multivalent forward reaction rate
$k_{r}$: Multivalent reverse reaction rate
$K_{A}$: Fundamental equilibrium constant
$k_{A}$: Individual multivalent attachment rate
$k_{D}$: Individual multivalent detachment rate
$\kappa_{A}$: Experimental multivalent attachment rate
$\kappa_{D}$: Experimental multivalent detachment rate
$\alpha$: Experimental power law exponent
$\beta$: Simulation power law exponent
$$k_{B}$$: Boltzmann constant
$$\mu_{b}$$: Chemical potential
$S$: Separation distance between receptor and ligand
*L*: Equilibrium bond length
$\kappa$: Hookean spring constant
$F_{sp}$: Hookean spring force of a biomolecular bond
$$C_{W}$$: Wall concentration
$\left[ B \right]$: Bound particle density
$P_{A}$: Monte Carlo Probability of Attachment
$P_{D}$: Monte Carlo Probability of Detachment
$t_{B}$: Time an NP is bound
$t_{ref}$: Reference time for unit consistnecy
$F_{sh}$: Shear force on a sphere
